# Apiin Promotes Healthy Aging in *C. elegans* Through Nutritional Activation of DAF-16/FOXO, Enhancing Fatty Acid Catabolism and Oxidative Stress Resistance

**DOI:** 10.3390/ijms262411888

**Published:** 2025-12-10

**Authors:** Yimin Qian, Xuebin Ding, Xinping Guo, Nan Bian, Ying Chen, Shaoyu Han, Wu Song, Lin Wei, Shuang Jiang

**Affiliations:** 1 College of Basic Medicine, Changchun University of Traditional Chinese Medicine, Changchun 130117, China; qian31aug@163.com (Y.Q.); five841110@126.com (W.S.); 2 Yantai Center for Disease Control and Prevention, No. 17 Fuhou Road, Laishan District, Yantai 264003, China; ytcdcdxb@126.com; 3 College of Traditional Chinese Medicine, Changchun University of Traditional Chinese Medicine, Changchun 130117, China; bjs605567605@163.com (N.B.); chenying66323@163.com (Y.C.); 4 Department of Statistical Science, University College London, 1-19 Torrington Place, London WC1E 6BT, UK; shaoyu.han.25@ucl.ac.uk

**Keywords:** apiin, flavonoids, *Caenorhabditis elegans*, anti-aging, antioxidant, metabolites, Daf-16/FOXO pathway

## Abstract

Apiin, a natural flavonoid sourced from parsley, demonstrates antioxidant properties; however, its specific anti-aging effects have yet to be investigated in *Caenorhabditis elegans* (*C. elegans*). This research utilized *C. elegans* models to examine the anti-aging effects of apiin and the underlying mechanisms. The findings indicated that 100 μg/mL apiin extended the mean lifespan of *C. elegans* by 26.70%. Furthermore, apiin improved age-related characteristics in *C. elegans*, such as reducing intestine lipofuscin accumulation and increasing head thrashes and body bends. Additionally, apiin significantly improved stress resistance under thermal, ultraviolet, and oxidative stress conditions. Transcriptomic analysis revealed that apiin induced the differential expression of genes related to fatty acid metabolism, lipid catabolism, and oxidoreductase activity in *C. elegans*. Metabolomic data further corroborated the modulation of fatty acid metabolic processes by apiin. Biochemical assays, including lipid staining, triglyceride quantification, and measurements of antioxidant enzyme activity, demonstrated a decrease in lipid content and an enhancement in antioxidant capacity in *C. elegans* treated with apiin. Moreover, apiin promoted the nuclear translocation of DAF-16 and upregulated key longevity-associated genes, including *sod-3*, *hsp-12.6*, *mtl-1*, and *ech-9*. These results indicate that apiin mitigates aging in *C. elegans* through mechanisms involving the activation of DAF-16 and the regulation of lipid metabolism and oxidative stress responses. Our findings underscore the potential of apiin as a natural therapeutic agent for aging and associated metabolic disorders.

## 1. Introduction

Aging is characterized by the progressive decline in physiological integrity and function, resulting in increased susceptibility to numerous age-related pathologies, such as cardiovascular diseases, neurological disorders, and metabolic disorders. This escalating issue has emerged as a significant public health challenge requiring immediate attention [1,2,3]. The free radical theory of aging, a fundamental concept in the discipline, asserts that the deterioration of function associated with aging is chiefly due to the buildup of oxidative damage induced by reactive oxygen species (ROS) [4]. Consequently, strategies aimed at enhancing ROS clearance or augmenting endogenous antioxidant systems represent prospective therapeutic approaches to mitigate the aging process and decelerate functional decline. In clinical practice, numerous anti-aging medications, including rapamycin, metformin, and senolytics, are often unsuitable for long-term use due to their potential to cause serious side effects [5,6,7]. Exogenous antioxidant therapies are crucial for combating aging, with naturally derived active compounds from plants being a primary focus in the formulation of anti-aging pharmaceutical agents [8,9]. The antioxidant curcumin extends the lifespan of *C. elegans* through the MAPK signaling pathway and has protective effects on dopaminergic neurodegeneration, also extending the lifespan of fruit flies [10,11]. Moreover, ergothioneine, an extract derived from Lion’s Mane mushroom, exhibits antioxidant and anti-inflammatory effects in mice, extending their average lifespan by 21% [12].

Increasing research indicates that age-related dyslipidemia, along with alterations in lipid signaling, is closely associated with cellular senescence, chronic inflammation, and oxidative damage, which are acknowledged as fundamental mechanisms of aging and determinants of lifespan. Interestingly, the lifespan-extending and health-enhancing effects of compounds such as crocin, phlorizin, butein, and ginsenoside are facilitated by the modulation of lipid metabolism [13,14,15,16]. Aberrant lipid accumulation induces oxidative stress through lipid peroxidation, thereby accelerating both cellular and organismal aging processes [17]. The aging process disrupts lipid metabolism, thus leading to several age-related illnesses, including atherosclerosis. Consequently, targeting lipid metabolic pathways has attracted growing interest as a potential therapeutic strategy to delay aging and extend healthspan [18]. Plant-derived natural compounds exhibit significant relevance in this domain owing to their multi-target regulatory functions and minimal toxicity. Total saponins from Panax japonicus (TSPJ) prevent obesity and regulate cholesterol levels in aging rats by modulating gut flora and enhancing microbial metabolism [19]. Hesperidin may similarly mitigate aortic inflammation and oxidative stress by diminishing the formation of advanced glycation end products (AGEs) in perivascular adipose tissue (PVAT) [20]. Quercetin has demonstrated significant lipid-lowering effects in *C. elegans*, alongside enhanced resistance to oxidative stress and extended lifespan [21]. These investigations further substantiate the promising potential of identifying anti-aging medicines that target lipid metabolism derived from flavonoid bioactive components.

Apiin (apigenin 7-O-[β-D-apiosyl-(1→2)-β-D-glucoside]), a natural flavonoid derived from medicinal plants like parsley, chamomile, and Sedum caeruleum, demonstrates significant antioxidant and lipid-lowering activities [22,23,24]. Additionally, it exhibits a variety of other pharmacological effects, including anti-inflammatory, antiviral, anticancer, and blood pressure-regulating properties. Its elevated thermal stability enables it to serve as an effective stabilizer and delivery medium for apigenin glycosides in food systems [25]. As a disaccharide glycoside of apigenin, apiin improves cell viability and decreases lipid peroxidation in yeast subjected to oxidative stress, while also mitigating the hepatotoxicity risks associated with analogues such as quercetin [26]. Research has validated its efficacy in reducing hyperglycemia and oxidative stress in diabetic conditions [24,27]. Investigations integrating network pharmacology with experimental validation demonstrated that apiin is crucial to the anti-atherosclerosis properties of *Ziziphora clinopodioides* Lam., chiefly by modulating oxidative stress, lipid metabolism, and inflammation [28]. Apiin’s distinctive multifunctional characteristics and the considerable plasticity of its molecular structure establish a robust basis for the formulation of novel anti-aging medicines. Due to its promising profile of diverse biological activity and safety characteristics, apiin warrants further investigation as a candidate substance in the realm of anti-aging and health maintenance.

The highly conserved DAF-16/FOXO pathway in *C. elegans*, which is directly homologous to mammalian FOXO, thus presents an ideal model for investigating the anti-aging mechanisms of the flavonoid apiin, with implications for higher organisms [29,30,31]. As a natural flavonoid glycoside, apiin functions as both a metabolic precursor to apigenin and a compound with intrinsic bioactivity. Its structure, featuring an apigenin aglycone for multi-target engagement and a unique apiose sugar for specificity, may enable synergistic, multi-pathway regulation [32,33,34,35,36,37]. This study aims to systematically evaluate the biological effects of apiin on aging, utilizing *C. elegans* as a model organism. We comprehensively validated apiin’s anti-aging efficacy by measuring *C. elegans* lifespan, motility, lipofuscin accumulation, and other aging indicators, in conjunction with assessments of in vitro and ex vivo antioxidant activities. Concurrently, transcriptomic and metabolomic analyses will be conducted to elucidate the key molecular pathways through which apiin modulates lipid metabolism and antioxidant activity. In conclusion, this study aims to establish apiin as a novel natural anti-aging agent, providing a robust theoretical foundation and experimental evidence for its future development into a safe, low-toxicity, and highly effective anti-aging nutritional supplement.

## 2. Results

### 2.1. Apiin Extends C. elegans Lifespan

**Figure 1:**
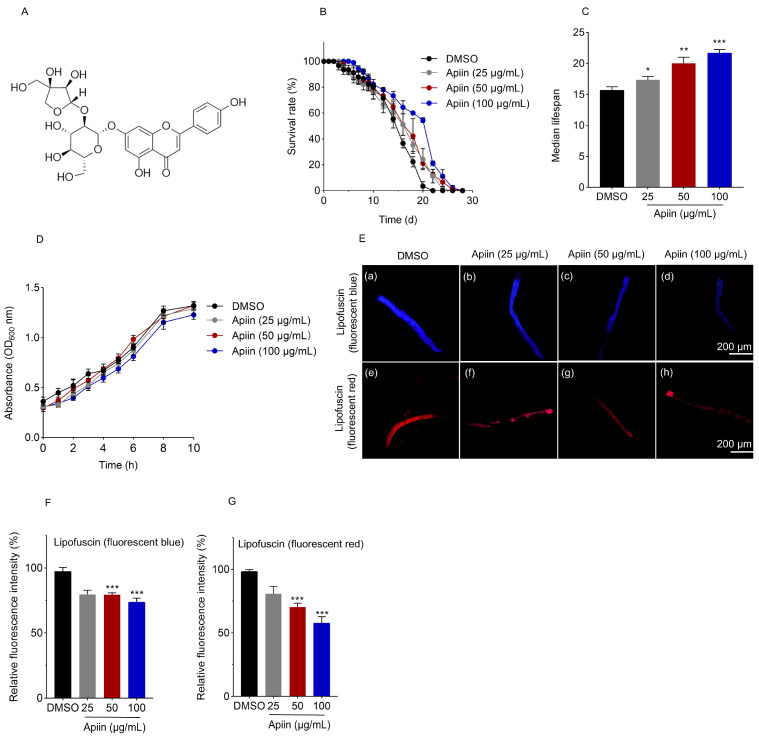
Effect of apiin on the lifespan of wild-type N2 *C. elegans*. (**A**) The structural formula of apiin. (**B**) The influence of DMSO and varying doses of apiin (25, 50, 100 μg/mL) on the lifespan of *C. elegans* was examined (Data were gathered from three biological replicates, with a total of 150 *C. elegans* per group divided into three dishes). (**C**) The median statistics regarding the impact of apiin at different concentrations (25, 50, 100 μg/mL) on the lifespan of *C. elegans*. (**D**) Growth trajectories of OP50 were analyzed in the presence of DMSO alongside various concentrations of apiin (25, 50, 100 μg/mL). (**E**–**G**)The quantification of lipofuscin accumulation in *C. elegans* at Day 5 of adulthood was conducted using blue autofluorescence (Ex/Em 340/430 nm) and red autofluorescence (Ex/Em 546/600 nm)**.** (**a**–**d**) and (**e**–**h**) illustrate the blue and red autofluorescence of lipofuscin in nematode intestines subjected to various concentrations of apiin (0, 25, 50, 100 μg/mL), respectively. Statistical significance was determined via one-way ANOVA with Tukey’s post hoc test for multiple comparisons (**C**,**D**,**F**,**G**), and via Kaplan–Meier survival analysis with the log-rank test (**B**). *p* < 0.05 was considered statistically significant (* *p* < 0.05, ** *p* < 0.01, *** *p* < 0.001).

To investigate the potential anti-aging effects of apiin (Figure 1A), wild-type N2 C. elegans were treated with concentrations of 25, 50, and 100 μg/mL, selected based on a preliminary effective range of 20–100 μg/mL, and lifespan was assessed. Lifespan data showed that apiin supplementation significantly extended the lifespan of *C. elegans* at all tested concentrations. Treatment with 100 μg/mL apiin increased both the mean lifespan and median lifespan by nearly 27% and 36%, respectively (Figure 1B,C). The growth of the *E. coli* OP50, the food source of *C. elegans*, was unaffected by apiin exposure (Figure 1D), indicating that its longevity-promoting effect does not stem from actions on OP50 growth or metabolism. Lipofuscin accumulation serves as a reliable biomarker for *C. elegans* aging. The red autofluorescence lipofuscin accumulates linearly with aging, while the blue lipofuscin increases sharply prior to death [38]. The result suggested that apiin treatment at 50 and 100 μg/mL significantly reduced lipofuscin accumulation (Figure 1E–G). Taken together, apiin effectively extended lifespan and attenuated lipofuscin accumulation in *C. elegans*.

### 2.2. Healthspan Profile of Apiin-Treated C. elegans

**Figure 2:**
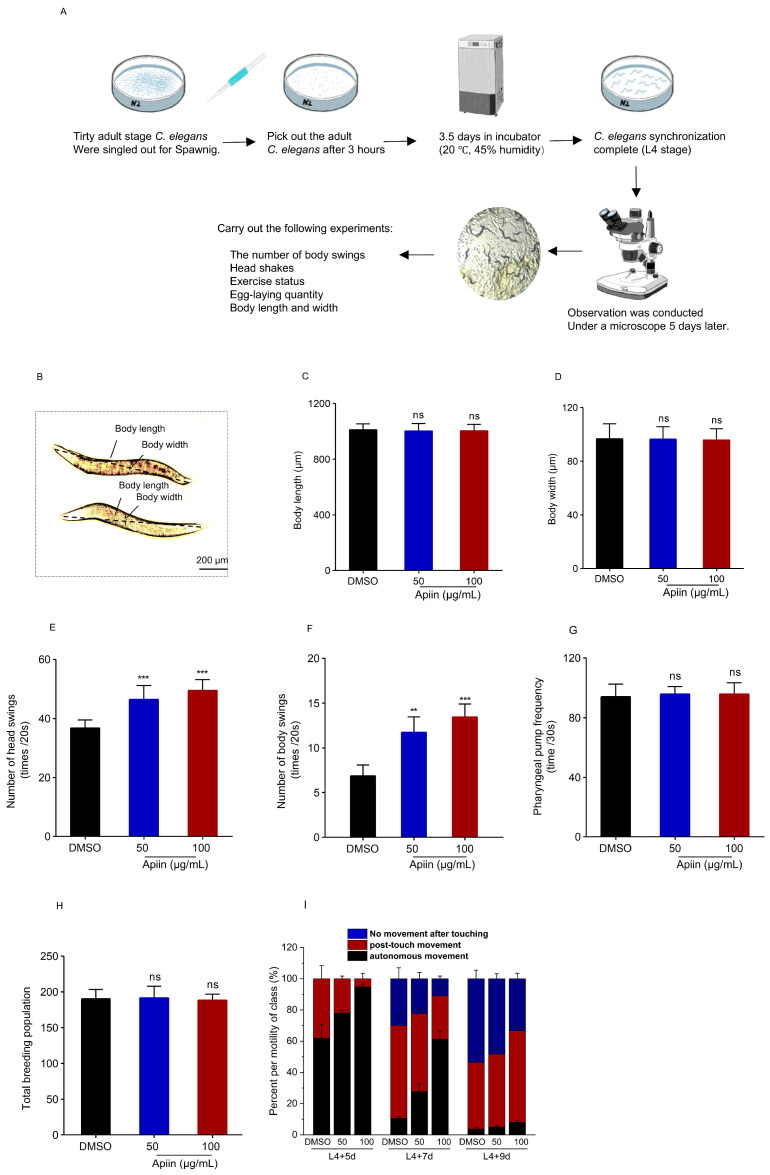
Effect of apiin on the health status of wild-type N2 *C. elegans*. (**A**) Flowchart of the experiment. (**B**–**D**) Impact of apiin on the body length and width of *C. elegans*. (**E**,**F**) Influence of apiin on the incidence of head swings and body swings in *C. elegans*. (**G**) The examination of how apiin affects the frequency of pharyngeal pumping in *C. elegans* is conducted. (**H**) Discussion on the influence of apiin on the reproductive functions of *C. elegans* is presented. (**I**) The assessment of apiin’s effects on the three motility states of *C. elegans* across three different time intervals is performed. Statistical analyses were conducted using one-way analysis of variance (ANOVA) followed by post hoc tests. *p* < 0.05 was considered statistically significant (** *p* < 0.01, *** *p* < 0.001). ‘ns’ denotes no significant difference.

The effects of apiin on *C. elegans* body shape, reproductive capacity and locomotor activity were assessed according to the flowchart (Figure 2A). The results showed that apiin treatment had little effect on body length or width (Figure 2B–D). The head swing and body bending frequency increased within a 20 s interval in apiin-treated *C. elegans*, compared to that in DMSO-treated *C. elegans* (Figure 2E,F). Additionally, pharyngeal pumping frequency, which responds to feeding status, was unaffected by apiin intake (Figure 2G). Lifespan extension has been associated with diminished reproductive output. [39,40]. Therefore, total production of eggs laid was measured from 1 to 5 days post-adulthood. No significant difference was observed compared to the DMSO-treated control group, indicating that apiin-mediated lifespan extension did not depend on fertility inhibition (Figure 2H). To further investigate the promoting effect of apiin on the movement ability, locomotion was analyzed in *C. elegans* at three aging time points: 8 days (Day 5 of adulthood), 10 days (Day 7 of adulthood), and 12 days (Day 9 of adulthood) (Figure 2I). The data showed that on Day 9 of adulthood, untreated worms primarily exhibited an absence of movement upon stimulation. Treatment with 50 μg/mL Apiin treatment significantly increased autonomous locomotion by 36.36% compared to controls. Collectively, these results demonstrated that apiin enhanced locomotor capacity, particularly in aged *C. elegans*.

### 2.3. Apiin Enhances C. elegans Antioxidant Capacity and Reduces Lipid Accumulation

**Figure 3:**
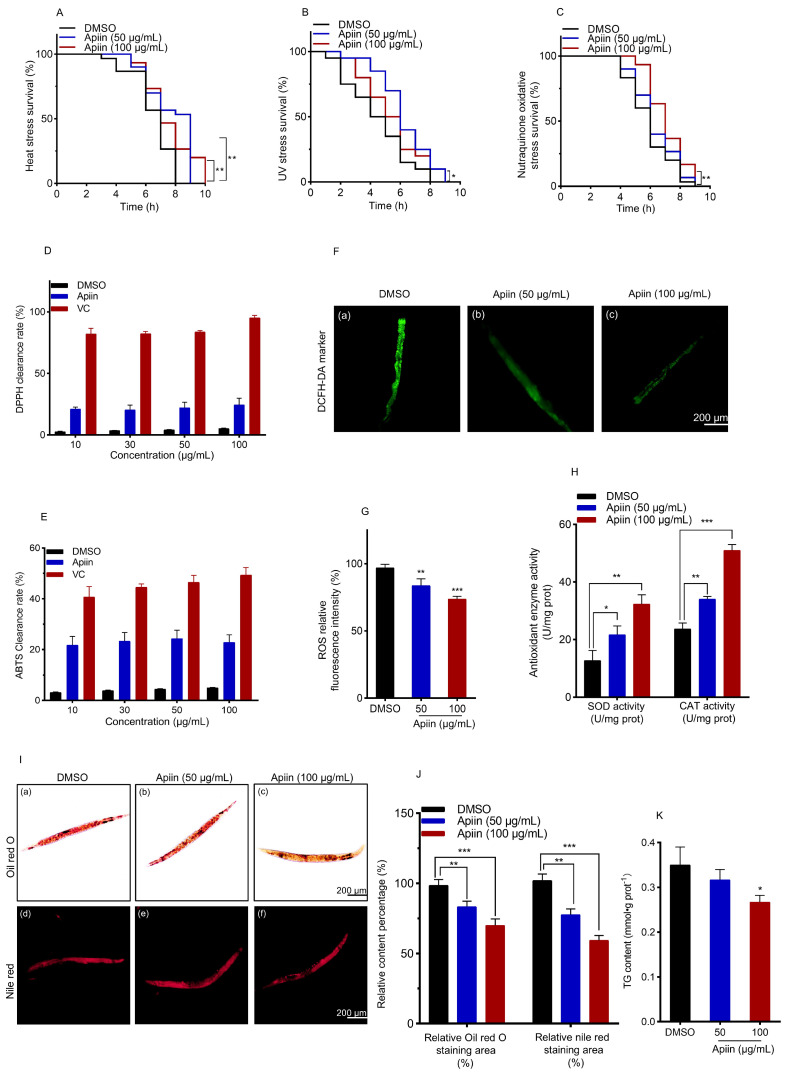
Effects of apiin on antioxidant activity and lipid metabolism in *C. elegans*. (**A**–**C**) Apiin (50 and 100 μg/mL) was investigated for its impact on the lifespan of *C. elegans* when subjected to heat stress at 37 °C, ultraviolet radiation, and oxidative stress caused by nutraquinone (Data are based on three biological replicates, with approximately 30 *C. elegans* in each). (**D**,**E**) The capacity for clearance of DPPH and ABTS was evaluated at varying concentrations of apiin (10, 30, 50, and 100 μg/mL). (**F**,**G**) The influence of apiin concentrations (50 and 100 μg/mL) on the accumulation of ROS in *C. elegans* was assessed. (**a**), (**b**) and (**c**) represent fluorescence images of ROS accumulation in nematodes treated with 0, 50 and 100 μg/mL apiin, respectively. (**H**) The impact of apiin concentrations (50 and 100 μg/mL) on the activities of SOD and CAT enzymes in *C. elegans* was also analyzed. (**I**,**J**) The effects of different concentrations of apiin (50 and 100 μg/mL) on Oil Red O (ORO) staining and Nile Red (NR) staining were examined. (**a**–**c**) and (**d**–**f**) represent microscopic observations of nematodes treated with apiin, which were stained with ORO and NR at concentrations of 0, 50, and 100 μg/mL, respectively. (**K**) A quantitative analysis of triglycerides was conducted to assess the effects of various concentrations of apiin (50 and 100 μg/mL). Statistical significance was determined via one-way ANOVA with Tukey’s post hoc test for multiple comparisons (**D**,**E**,**G**,**H**,**J**,**K**), and via Kaplan–Meier survival analysis with the log-rank test (**A**–**C**). *p* < 0.05 was considered statistically significant (* *p* < 0.05, ** *p* < 0.01, *** *p* < 0.001).

To investigate the impact of apiin on stress resistance, *C. elegans* (Day 5 of adulthood) were subjected to thermal stress, UV stress and juglone-induced oxidative stress assays (Figure 3A–D). Our results demonstrated that apiin (50 and 100 μg/mL) significantly enhanced thermotolerance and oxidative stress resistance, as evidenced by markedly improved survival rates under heat challenge at 37 °C and juglone exposure. Similarly, apiin treatment also increased resistance to UV stress. To evaluate the radical scavenging activity of apiin in vitro, DPPH and ABTS^+^ radical scavenging assays were performed. Apiin (10–100 μg/mL) exhibited significant radical scavenging capacity. At 100 μg/mL, DPPH and ABTS^+^ radical scavenging rates reached 24.47 ± 5.45% and 22.86 ± 2.91%, respectively (Figure 3E,F). To further assess apiin’s ability to mitigate oxidative damage in vivo, reactive oxygen species (ROS) accumulation was measured, and the activities of superoxide dismutase (SOD) and catalase (CAT) were determined. The data showed that apiin led to a decrease in ROS fluorescence intensity, indicating a decrease in ROS accumulation in *C. elegans*. Specifically, mean fluorescence intensity declined by 13.33% and 23.33% following treatment with 50 and 100 μg/mL apiin, respectively. Furthermore, apiin treatment significantly elevated both SOD and CAT activities (Figure 3G,H). Collectively, these findings demonstrated that apiin effectively reduced ROS accumulation in *C. elegans* and exhibited potent antioxidant activity.

Given the antioxidant capacity of apiin in aged *C. elegans*, we further investigated its potential effects on lipid metabolism (Figure 3I–K). Quantitative analysis using ORO and NR staining demonstrated that treatment with 100 μg/mL apiin significantly reduced fat accumulation in aged *C. elegans* by 29.05% and 41.83%, respectively. Furthermore, the same treatment decreased triglyceride (TG) content in aged *C. elegans*. These findings suggest that apiin alleviates age-related dysregulation of lipid metabolism. In conclusion, apiin enhances both antioxidant activity and lipid metabolism in *C. elegans*.

### 2.4. RNA Sequencing Profile

**Figure 4:**
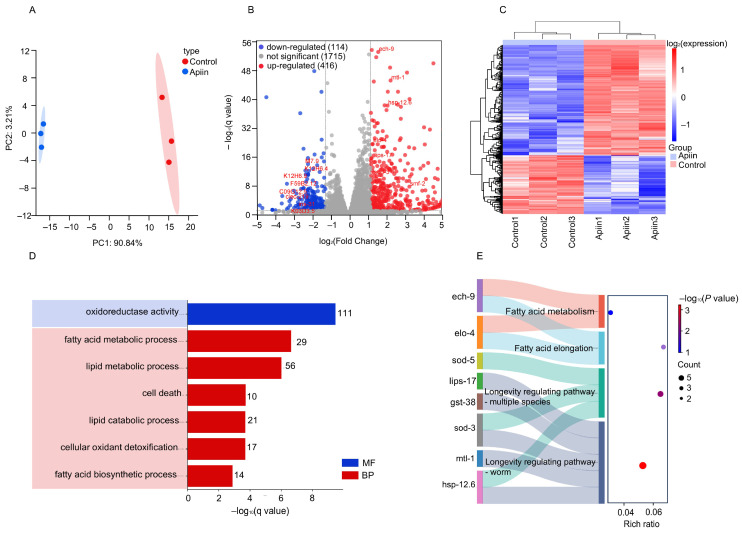
The RNA sequence and enrichment analysis were conducted for the Apiin group (100 μg/mL apiin) and the Control group. (**A**) Principal Component Analysis (PCA) is used. (**B**) A Volcano plot depicting genes that are expressed variably (*q* < 0.05, |log_2_FC| > 1.2). (**C**) A heatmap clustering these variably expressed genes. (**D**) Gene Ontology (GO) pathway assessment emphasizing the most crucial components of molecular function (MF) and biological process (BP). (**E**) KEGG pathway evaluation of genes that are expressed variably. In this KEGG analysis, the dimensions of the circles reflect the quantity of differentially expressed genes, with color coding based on −log_10_ (*p* value). The X-axis represents the enrichment factor value.

To elucidate the potential mechanisms underlying apiin-mediated lifespan extension in *C. elegans*, RNA-seq analysis was performed. Principal component analysis (PCA) revealed distinct clustering between the control group and the 100 µg/mL apiin-treated group (Figure 4A). Differential mRNA expression analysis identified 590 significantly differentially expressed genes (DEGs, *q* < 0.05, |log_2_FC| > 1.2), comprising 416 upregulated and 174 downregulated genes (Figure 4B,C). Gene Ontology (GO) enrichment analysis indicated predominant enrichment of oxidoreductase activity terms in the MF category. The Biological Process (BP) category showed significant enrichment for processes including fatty acid metabolic process, lipid metabolic process, lipid catabolic process, cellular oxidant detoxification and fatty acid biosynthetic process (Figure 4D). Kyoto Encyclopedia of Genes and Genomes (KEGG) pathway analysis highlighted four significantly altered pathways, including fatty acid metabolism, fatty acid elongation, longevity regulating pathway-multiple species and longevity regulating pathway-worm (Figure 4E). Based on the differentially expressed genes identified through KEGG enrichment analysis presented in Figure 4E, we constructed a gene association network diagram (refer to Table S2 and Figure S2). Notably, *daf-16* emerged as the first gene when the genes were sorted by degree. These results suggest that apiin may extend the lifespan of *C. elegans* by activating antioxidant enzymes and regulating lipid metabolism, all of which may be related to *daf-16*.

### 2.5. The Anti-Aging Effect of Apiin Is Mediated by DAF-16

**Figure 5:**
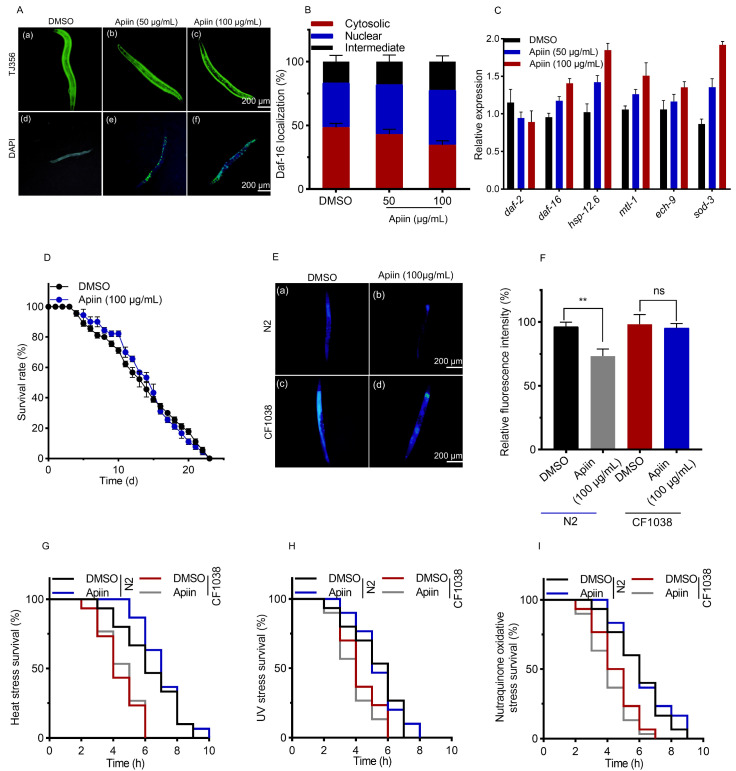
Apiin influences the lifespan and health of *C. elegans* by regulating DAF-16. (**A**,**B**) The translocation of DAF-16::GFP into the nucleus was noted in TJ356 *C. elegans* upon apiin treatment (n = 20). (**C**) The expression levels of mRNA corresponding to DAF-16 target genes were evaluated in wild-type N2 *C. elegans* subjected to 100 μg/mL apiin. (**A**): (**a**–**c**) and (**d**–**f**) depict fluorescence images illustrating nuclear displacement in TJ356 nematodes subjected to treatments of 0, 50, and 100 µg/mL apiin, with (**d**–**f**) including DAPI staining for enhanced visualization. (**D**) The survival curves of CF1038 [*daf-16*(mu86)I] *C. elegans* undergoing treatment with 100 μg/mL apiin in normal conditions were examined. (**E**,**F**) The accumulation of lipofuscin was measured in wild-type N2 and CF1038 mutants treated with 100 μg/mL apiin, respectively. (**E**): (**a**,**b**) and (**c**,**d**) depict blue fluorescence of intestinal lipofuscin in N2 and CF1038 nematodes, respectively, after treatment with 0 and 100 µg/mL apiin. (**G**–**I**) The impact of apiin on the lifespan of N2 and CF1038 mutants during heat stress at 37 °C, ultraviolet stress, and oxidative stress triggered by nutraquinone was assessed (data were obtained from three biological replicates, with approximately 30 *C. elegans* per replicate). Statistical significance was determined via one-way ANOVA with Tukey’s post hoc test for multiple comparisons (**B**–**D**,**F**), and via Kaplan–Meier survival analysis with the log-rank test (**G**–**I**). *p* < 0.05 was considered statistically significant (** *p* < 0.01). ‘ns’ denotes no significant difference.

In *C. elegans*, nuclear translocation of the FOXO transcription factor DAF-16 activates protective genes, enhancing stress resistance and metabolic homeostasis to extend lifespan [41,42]. To determine whether apiin promoted nuclear translocation of DAF-16, the localization of DAF-16 was observed in transgenic *C. elegans* strain TJ356 (DAF-16::GFP). Compared to the control group, treatment with 100 µg/mL apiin resulted in a significant increase (22.86 ± 5.96%) in nuclear localization and a significant decrease (28.27 ± 4.72%) in cytoplasmic localization, indicating that apiin promoted nuclear accumulation of DAF-16 (Figure 5A,B). Furthermore, apiin significantly upregulated the mRNA expression levels of key DAF-16-regulated genes associated with antioxidant stress response and lipid metabolism, including *sod-3*, *hsp-12.6*, *mtl-1* and *ech-9* (Figure 5C). This finding is consistent with the results of differential gene expression analysis by RNA-seq, as shown in the volcano plot in Figure 4B.

To further investigate whether apiin extended lifespan via the DAF-16 pathway, functional validation was performed by using the mutant *C. elegans* strain CF1038 [*daf-16*(mu86)I]. We first assessed the effects of apiin on the survival and accumulation of lipofuscin in CF1038 mutants and N2 *C. elegans* (Figure 5D–F). The results showed that treatment with 100 μg/mL apiin did not significantly extend the lifespan of CF1038 mutants, in contrast to the significant lifespan extension observed in N2 *C. elegans*. Additionally, apiin had no significant effect on reducing lipofuscin accumulation in CF1038 mutants. We further examined the impact of apiin on stress resistance in CF1038 mutants by conducting heat stress, UV stress and nutraquinone-induced oxidative stress assays. Apiin treatment failed to enhance the stress resistance of CF1038 mutants (Figure 5G–I). Collectively, these data demonstrated that DAF-16 was a critical mediator of apiin-induced longevity promotion in *C. elegans*.

### 2.6. Metabolomics Analysis Revealed the Mechanism of Action of Apiin on Fatty Acid Catabolism

**Figure 6:**
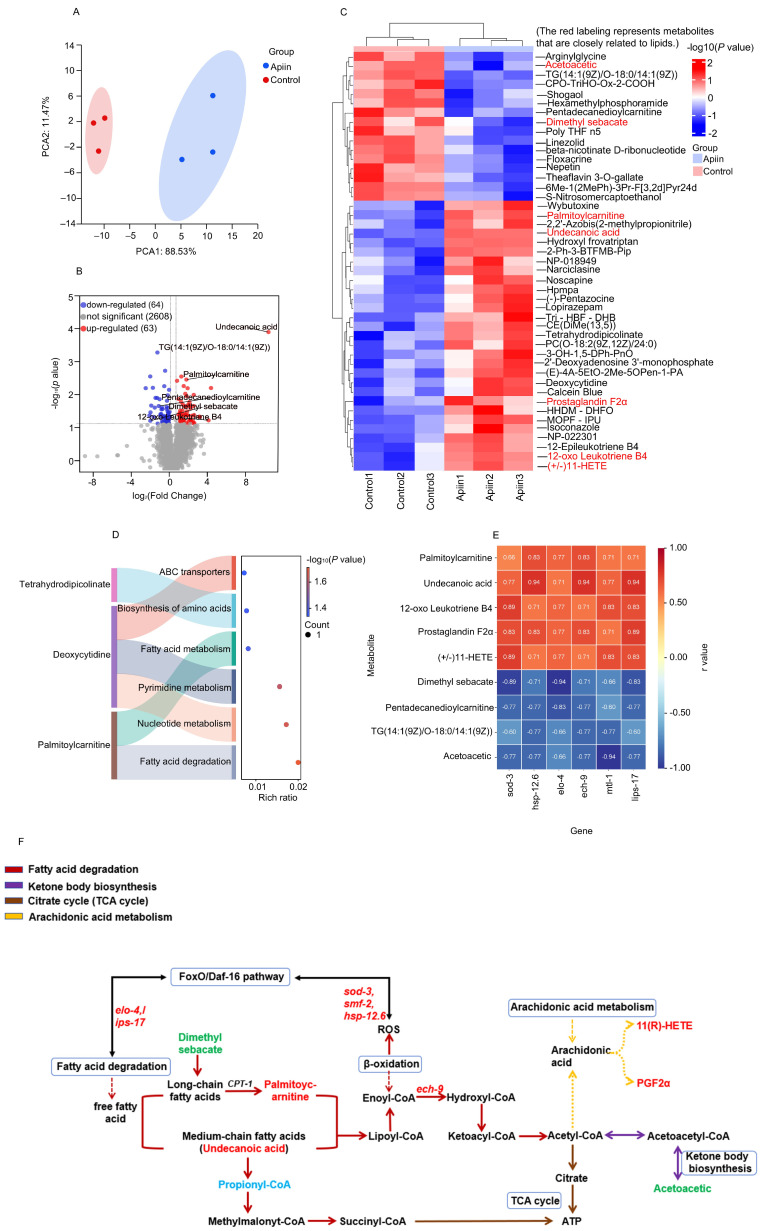
Metabolite analysis of apiin group (100 μg/mL apiin) and Control group. (**A**) Score plot for PCA (**B**) Volcano plot comparing the Control and Apiin groups. (**C**) Clustering heat map illustrating differential metabolites (*p* < 0.05, |log_2_FC| > 1.2). (**D**) Results of KEGG pathway enrichment based on various metabolites. (**E**) Relationship between metabolites and genes related to antioxidants. (**F**) Impact of apiin treatment on the metabolic pathways in *C. elegans*. Coloured lines denote pathways associated with the figure legend. Genes and metabolites labeled in red indicate significant upregulation, while those in green denote significant downregulation. Blue metabolites represent no significant change. Solid lines signify direct promotion or direct association, whereas dashed lines indicate indirect promotion or association.

Metabolomics analysis was employed to investigate the key metabolites and mechanism of action underlying apiin-mediated lifespan extension in *C. elegans*. PCA revealed distinct clustering of the control group with the 100 µg/mL apiin-treated group (Figure 6A). We identified 127 significantly differential metabolites (63 upregulated, 64 downregulated), including 45 exhibiting substantial differences (*p* < 0.05 and |log_2_FC| > 1.2) (Figure 6B,C). KEGG enrichment analysis indicated that apiin primarily affected pathways related to fatty acid degradation, fatty acid metabolism and ABC transporters (Figure 6D). To elucidate functional relationships, Pearson correlation analysis assessed the association between metabolite changes and differential expression of antioxidant/anti-aging-related genes following apiin exposure, aiming to uncover potential anti-aging pathways (Figure 6E,F). Mechanistically, the upregulation of *ech-9* expression showed strong positive correlations with palmitoylcarnitine (r = 0.83) and undecanoic acid (r = 0.94), metabolites facilitating mitochondrial fatty acid transport for β-oxidation. Concurrently, *ech-9* upregulation correlated negatively with triglyceride levels (r = −0.77). Furthermore, oxidative stress markers 12-oxo-leukotriene B4 and (±)11-HETE correlated positively with downstream antioxidant genes of *daf-16*, such as *sod-3*. This suggested oxidative stress activates the *daf-16* pathway, upregulating *sod-3* to scavenge ROS generated during β-oxidation. The *daf-16* pathway also coordinately regulated genes like *lipl-17* and *hsp-12.6* to enhance medium-chain and long-chain fatty acid degradation. This integrated β-oxidative-antioxidative response ultimately mediates the lifespan-extending effects of apiin. In summary, apiin promotes lifespan by activating *daf-16*-regulated target genes (*lipl-17*, *hsp-12.6*, *elo-4*), enhancing degradation of long-chain and medium-chain fatty acids. This facilitates the metabolism of palmitoylcarnitine and undecanoic acid, which enter β-oxidation via *ech-9* upregulation. Superoxide dismutase 3 (SOD-3), encoded by the *sod-3* gene, is a critical antioxidant enzyme that counteracts ROS generated during β-oxidation. This highlights the significant relationship between fatty acid degradation and antioxidant defense mechanisms. Consequently, metabolomics analyses indicate that apiin extends lifespan by enhancing fatty acid metabolism and alleviating oxidative stress through a synergistic interplay between β-oxidation and antioxidant systems.

## 3. Discussion

As research on the mechanisms of aging progresses, intervention strategies have increasingly focused on dietary phytochemicals with antioxidant and anti-inflammatory properties [43,44]. The ability of such compounds to alleviate oxidative stress and low-grade inflammation underscores their potential as anti-aging therapeutics [45,46]. Notable examples include polyphenolic flavonoids such as salicin, kaempferol, and baicalein, which have been shown to extend lifespan and improve healthspan in models ranging from *C. elegans* to mammals [47,48,49]. In line with these findings, our study identifies apiin, a flavonoid derived from parsley, as a novel anti-aging agent in *C. elegans*. We demonstrate for the first time that apiin not only prolongs lifespan but also enhances healthspan in this model. Genetic analyses using mutant strains, along with transcriptomic and metabolomic approaches, indicate that apiin-mediated longevity requires DAF-16/FOXO activation, which is further supported by the observed nuclear translocation. These results establish apiin as a natural activator of conserved longevity pathways and highlight the potential of plant-derived flavonoids in the development of targeted anti-aging interventions.

Prior studies have shown the significant antioxidant properties of apiin in vitro. We investigated its possible effects in vivo by using *C. elegans* as a model organism. Apiin was shown to increase the average lifespan of wild-type N2 *C. elegans* by 26.70%, while decreasing the accumulation of ROS and lipofuscin, without any detrimental effects on growth, development, or reproductive capacity. These data corroborate the function of apiin as an in vivo antioxidant and a facilitator of lifespan, aligning with an established understanding of its antioxidant characteristics. To determine whether apiin prolongs lifespan by inhibiting bacterial proliferation, we introduced apiin at different doses into LB medium containing *E. coli* OP50. Apiin did not suppress the proliferation of *E. coli* OP50 at these concentrations (the findings did not indicate any effect within ten hours), suggesting that apiin is unlikely to prolong the lifetime of *C. elegans* via bacterial growth inhibition. Considering the pivotal function of the conserved transcription factor DAF-16/FOXO in modulating lifespan, stress resistance, and metabolism, we examined its role in the action mechanism of apiin [50]. The apiin-mediated lifespan extension was nullified in *daf-16* mutants but maintained in N2 controls, suggesting that the effects of apiin rely on DAF-16/FOXO. Further tests revealed that apiin promotes the nuclear translocation of DAF-16 and increases the expression of downstream antioxidant genes (including *sod-3*, *mtl-1*, and *hsp-16.2*) in aged *C. elegans*. The suppression of *daf-16* expression via RNA interference eliminated apiin-induced stress resistance. These results collectively demonstrate that DAF-16/FOXO is a crucial mediator of the ability of apiin to prolong longevity and provide antioxidant benefits in *C. elegans* [51].

Disruptions in lipid homeostasis, along with conditions that promote oxidative stress, lead to a reduction in organismal lifespan [52,53]. Research has indicated that reactive aldehyde species produced by lipid peroxidation expedite the development of neurological disorders in the aging population [54,55]. Moreover, the buildup of oxidative stress, inflammation, and low-density lipoprotein (LDL) particles intensifies the progression of atherosclerosis [56,57]. Thus, therapies aimed at lipid metabolism constitute a viable approach for prolonging lifespan [58]. *C. elegans*, a prominent model organism in gerontology, has exhibited lifespan prolongation in response to many natural compounds. Interestingly, the lifespan-extending and health-enhancing benefits of substances such as crocin, phlorizin, butein, and ginseng saponins are facilitated by the modulation of lipid metabolism [13,14,16,59]. This study demonstrated that apiin administration markedly decreased lipid accumulation in aged *C. elegans*. This reduction was associated with decreased relative ROS levels and improved stress resilience. The age-specific lipid-lowering effect of apiin corresponds to its anti-aging effects, as both lipid accumulation and oxidative stress often increase with advancing age. Transcriptomic analyses revealed that the expression of antioxidant and lipid autophagy genes linked to DAF-16 was markedly elevated in apiin-treated *C. elegans*. The results of the GO analysis indicated a strong correlation of these genes with oxidoreductase activity and lipid metabolism, among other functions, whereas the results of the KEGG analysis specifically highlighted pathways linked to fatty acid metabolism and longevity regulation.

Research has shown that mitochondrial fatty acid oxidation promotes the aging process [60]. This research examines the mechanism through which apiin modulates metabolic pathways to prolong longevity. A metabolomics study revealed that apiin therapy markedly improved the breakdown of long-chain and medium-chain fatty acids. Mechanistic investigations revealed that apiin activates the DAF-16 pathway, resulting in the overexpression of genes such as *elo-4* and *lips-17*. This activation improves the conversion of long-chain fatty acids into palmitoylcarnitine and facilitates the breakdown of medium-chain fatty acids, such as undecanoic acid. As a result, these fatty acid derivatives enter the mitochondrial β-oxidation route, ultimately producing acetyl-CoA, which supplies cellular energy and precursors. Notably, this process is accompanied by ROS production. Apiin treatment concurrently induces the expression of antioxidant genes, such as *sod-3* (encoding superoxide dismutase SOD-3), *smf-2*, and *hsp-12.6*. Among these, SOD-3 effectively scavenges the ROS generated during β-oxidation, thereby maintaining cellular redox homeostasis and ensuring efficient fatty acid degradation and unimpeded energy metabolism [61,62]. Furthermore, the arachidonic acid metabolites PGF2α and 11(R)-HETE may synergize with the pro-fatty acid degradation and antioxidant effects of apiin through their immunomodulatory actions. Conversely, alterations in acetoacetic acid derived from β-oxidation warrant attention, as its increased utilization indicates accelerated rates of lipid degradation. These metabolic pathways, primarily focused on fatty acid degradation, promote ATP production, ensuring that sufficient energy is ultimately generated through the TCA cycle to maintain metabolic homeostasis in *C. elegans*. Importantly, downstream targets of *daf-16* regulate both long-chain and medium-chain fatty acid degradation pathways. They not only directly drive fatty acid β-oxidation but also increase cellular antioxidant capacity, effectively counteracting ROS accumulation. These findings confirm that apiin-mediated lifespan extension occurs through the DAF-16/FOXO pathway by enhancing antioxidant capacity and lipid regulatory activity in aged *C. elegans*.

To our knowledge, this study provides the first evidence that apiin extends the lifespan of *C. elegans* by enhancing its antioxidant capacity and modulating its lipid metabolism. These findings address the existing information gap concerning apiin bioactivity in invertebrate models and elucidate the processes underlying its lifespan-extending and health-promoting benefits. Nonetheless, certain limitations must be acknowledged, particularly in elucidating the functional relationship between essential metabolites and daf-16, as well as the precise regulatory mechanisms through which these metabolites affect longevity. Our study did not ascertain whether apiin exerts its effects in *C. elegans* via conversion to apigenin or in its glycosylated form. To bridge this gap, it is imperative to directly quantify the potential conversion of apiin into apigenin using liquid chromatography-mass spectrometry. Furthermore, the necessity of this metabolic step for longevity effects must be validated through genetic or pharmacological approaches. Establishing the broader significance of these findings will also necessitate validation in complementary mammalian models. This delineates the trajectory of our forthcoming research endeavors.

## 4. Materials and Methods

### 4.1. Reagents and Materials

The strains of *E. coli* OP50 and *C. elegans* (wild-type N2, TJ356, and CF1038) were obtained from the Center for Nematode Genetics (CGC) located in Minneapolis, MN, USA. Apiin (CAS# 26544-34-3; Yuanye, Shanghai, China) stock solutions in DMSO (Cat# D4540; Sigma-Aldrich, Shanghai, China) were diluted in NGM to achieve final concentrations of 25, 50, and 100 μg/mL (0.1% *v*/*v* DMSO). The control plates were treated with 0.1% DMSO alone. Details regarding the extraction and chemical characterization of apiin can be found in Figure S1. The subsequent reagents utilized in the experiments included agar, KH_2_PO_4_, Na_2_HPO_4_, and NaH_2_PO_4,_ which were sourced from Tianli Chemical Reagents Co., Ltd. (Tianjin, China). Sodium chloride was procured from CSNpharm (Chicago, IL, USA), and cholesterol was supplied by Solarbio (Beijing, China). Reagents already available in the laboratory included NaClO, CaCl_2_, NaOH, MgSO_4_, yeast extract, tryptone, and peptone. Levamisole hydrochloride (CAS#16595-80-5) was obtained from Shanghai Yuanye Biotechnology Co., Ltd. (Shanghai, China). The SOD and CAT Assay Kits, as well as the Reactive Oxygen Assay Kit, were obtained from Beyotime (Shanghai, China). The BCA Kit was used for laboratory inventories.

### 4.2. Culture and Treatment of C. elegans

*C. elegans* worms were grown in Nematode Growth Medium (NGM) at a temperature of 20 °C, with *E. coli* OP50 serving as the feeding source. The worms were synchronized either through sodium hypochlorite treatment or by selecting them after they had spawned. The methods employed for cultivation and maintenance were consistent with those outlined in the WormBook. The wild-type N2 strain was characterized for a range of parameters, from organismal phenotypes (lifespan, lipofuscin, mobility and reproductive capacity, stress tolerance tests, antioxidant capacity and lipid staining) to systemic molecular profiles (transcriptomics and metabolomics analyses). Subcellular localization of DAF-16 was assessed in the TJ356 strain [*daf-16*::gfp], whereas genetic validation of DAF-16-dependent effects was conducted through loss-of-function analysis in the CF1038 mutant [*daf-16*(mu86)I]. The worms were cultured in Petri dishes containing 50 and 100 µg/mL of apiin mixed into the NGM, and the surface was subsequently coated with OP50. These dishes were kept overnight at 20 °C before being used. Afterwards, *C. elegans* eggs were incubated in NGM at 20 °C for 3.5 days with OP50, DMSO, and apiin concentrations of 50 and 100 µg/mL to allow them to reach the adulthood. Following this period, experiments were performed to assess the impact of apiin on *C. elegans*. Student’s *t*-test was used for the statistical evaluation, and the data are expressed as the mean ± standard deviation (mean ± SD).

### 4.3. Lifespan Analysis

Experiments on lifespan were conducted at a temperature of 20 °C. A minimum of 150 synchronized adult N2 *C. elegans* worms were transferred onto NGM plates (60 mm in diameter) enriched with OP50. The day of synchronization was marked as Day 0 of the study. To prevent oviposition, *C. elegans* was transferred daily to fresh plates containing apiin or a control without it. Those worms that did not respond to mechanical stimulation were marked as deceased. The survival rate of *C. elegans* in each group was tracked, and the number of nematodes that died was recorded until every *C. elegans* in the group had perished. In the lifespan experiment of the genetic validation study, the CF1038 [*daf-16*(mu86)I] strain was used, following the same methodology as above.

### 4.4. Lipofuscin Assays

Synchronized N2 *C. elegans* worms were exposed to DMSO or apiin (50, or 100 μg/mL) 8 days to promote growth to Day 5 of adulthood. Afterwards, all worms were washed with M9 buffer three times to remove any residual bacteria. The worms were subsequently anesthetized using 25 mM levamisole hydrochloride and fixed on 2% agarose pads for fluorescence imaging. The fluorescence intensity of lipofuscin was measured with ImageJ (1.54g), utilizing three biological replicates, each containing 20 worms. In the lipofuscin assays for genetic validation experiments, the CF1038 [*daf-16*(mu86)I] nematode strain was used, following the same procedure as above.

### 4.5. Antibacterial Assay

A dilution of apiin in OP50 was prepared inside a 96-well microplate (SORFA, Deqing, China). One hundred microlitres of the OP50 suspension containing apiin was incubated at 37 °C and analysed using a microplate reader (Thermo Fisher Scientific, Waltham, MA, USA). The OD_600_ measurements were taken on an hourly basis until the ninth hour.

### 4.6. Body Length and Width Measurements

Synchronized adult N2 *C. elegans* worms were exposed to 50 and 100 µg/mL apiin for three days. Following this period, three individuals from each treatment group were randomly chosen and placed on slides, and anaesthetized using 25 mM levamisole (Yuanye, Shanghai, China). Once they became rigid, the specimens were observed and photographed for documentation purposes. Body length and width were measured under an Olympus X71 microscope (Olympus Co., Tokyo, Japan).

### 4.7. Head and Body Swinging Assays

Experiments involving head and body swinging were conducted by treating the synchronized adult N2 *C. elegans* worms with 50 and 100 μg/mL apiin for a period of 3 days. Body bending was defined as the performance of a sinusoidal S-curve, whereas the side-to-side movement of the head was referred to as head swinging. The rates of both head swinging and body bending were measured under a microscope during a 20-s observation period.

### 4.8. Reproduction Assays

The N2 worms were synchronized according to earlier methods, and three individuals at the adult stage were chosen from each tray, after which they were allowed to spawn for approximately three hours. The adults were removed every 24 h until egg production was complete, and the total number of hatched eggs was counted.

### 4.9. Pharyngeal Pumping Assay

Synchronized N2 *C. elegans* adults were treated with 50 and 100 µg/mL apiin for 8 days. The pharyngeal pumping activity of these *C. elegans* worms was quantified using a microscope for 30 s.

### 4.10. Stress Tolerance Tests

The N2 *C. elegans* worms were treated with DMSO and apiin at a concentrations of 50 or 100 µg/mL until they reached Day 5 of adulthood, and were then distributed into control and treatment cohorts of 90 individuals each. Afterwards, the nematodes were exposed to ultraviolet light at an energy level of 100 mJ to assess the efficacy of apiin in protecting against UV damage. Additionally, the worms were kept at 37 °C, and mortality was recorded on an hourly basis to determine apiin’s resistance to heat stress. The approach for grouping and standardization in the oxidative stress examination was similar to that of the heat stress assessment, whereby 90 *C. elegans* individuals from each group were placed onto NGM plates with nutraquinone at a final concentration of 500 μM; the plates were initially free of *C. elegans*. The counts of mortality, number of survivors, and survival rates for *C. elegans* were recorded hourly. The death toll was determined on the basis of the number of *C. elegans* worms that died, while the survival count was calculated from the mortality data. Throughout each experiment, a *C. elegans* worm was deemed deceased if it showed no response when touched with a platinum wire. Accidental deaths, including those caused by falls or climbing the walls, were not counted. Data were analysed using the log-rank test. In the stress tolerance tests of the gene validation experiment, the CF1038 [*daf-16*(mu86)I] strain was used, following the same procedure as above.

### 4.11. In Vitro Antioxidation Assay

The ability of apiin to scavenge free radicals was assessed using DPPH and ABTS assays, which validated its direct antioxidant potential. Previous studies have provided valuable insights into experimental methods [63,64]. A 0.3 mL sample was mixed with 2.7 mL of a 0.5 mM DPPH solution prepared in methanol, and the resulting mixture was allowed to stand in the dark for 20 min. Absorbance was measured at 519 nm by using a blank as the negative control. The clearance rate was calculated using the following formula, where A_0_ and A_S_ denote the absorbance values for the blank and samples, respectively. DPPH clearance activity (%) = (A_0_ − A_S_)/A_0_ × 100%

A mixture of 7.4 mM ABTS diammonium and 2.6 mM potassium perbisulfite (K_2_S_2_O_8_) was prepared and allowed to sit in the dark at room temperature for 16 h. After this period, the solution was diluted with phosphate buffer (pH 7.4) to obtain an absorbance reading of 0.700 ± 0.002 at 734 nm, thus creating the working solution of ABTS^+^. Afterwards, 0.1 mL of the sample was added to 3.9 mL of the ABTS^+^ solution, and the absorbance was measured at 734 nm following a 6-min reaction time, with A_0_ and A_S_ indicating the absorbance values for the blank and samples, respectively. ABTS clearance activity (%) = (A_0_ − A_S_)/A_0_ × 100%

### 4.12. Determination of ROS and Antioxidant Enzymes

Synchronized N2 *C. elegans* adults were treated with NGM supplemented with DMSO or apiin at concentrations of 50 and 100 µg/mL for 48 h. After this treatment, the adults were rinsed three times with M9 solution. Next, an active oxygen probe (DCFH-DA) was added at a dilution of 1:1000 and incubated at 20 °C for 25 min. Following this incubation period, the *C. elegans* worms were washed three times with M9 buffer and then placed on 2% agarose pads for imaging. The levels of reactive oxygen species (ROS) were measured at an excitation wavelength of 488 nm and an emission wavelength of 525 nm. Synchronized *C. elegans* adults were also treated with NGM supplemented with 0, 50, or 100 µg/mL apiin. A total of 1000 *C. elegans* adults were selected and rinsed with M9 buffer to remove any residual OP50. The supernatant was then collected through grinding, crushing, and centrifugation at 3000 rpm for 10 min in an ice bath. The concentrations of superoxide dismutase (SOD) and catalase (CAT) were determined following the protocols outlined in the commercial kits. The protein concentrations were subsequently measured using the BCA assay kit. Student’s *t*-test was employed for statistical analysis. The data are expressed as the mean ± SD.

### 4.13. NR Staining

NR staining was quantitatively analysed according to previously established methods. N2 *C. elegans* worms were briefly harvested in M9 buffer and subsequently fixed using 40% isopropanol for 3 min. To prepare the functional solution, 6 μL of NR stock solution (0.5 mg mL^−1^ in acetone) was mixed with 1 mL of 40% isopropanol. After fixation, the NR solution was added, and the worms were incubated at 20 °C in the dark for two hours. The NR staining solution was then removed, and the specimens were washed for 30 min with M9 solution, followed by three additional washes to eliminate any residual staining. Fluorescence measurements were taken and analysed using ImageJ, maintaining 20 *C. elegans* worms per set and three biological replicates.

### 4.14. ORO Staining

ORO staining was performed following previously established methods with slight modifications. N2 *C. elegans* worms were collected in M9 buffer and then fixed using 4% paraformaldehyde for 1 h. Afterwards, the samples were flash-frozen in liquid nitrogen for 5 min, followed by dehydration and fixation in 60% isopropanol for 15 min. The samples were incubated with ORO working solution (prepared by mixing ORO stock solution and ddH_2_O at a 3:2 ratio, *v*/*v*) at 37 °C for 4 h to facilitate staining. Any excess dye was removed by rinsing the samples with M9 buffer, after which the samples were observed under a fluorescence microscope. In each replicate, approximately 20 *C. elegans* individuals were analysed, and three biological replicates were maintained.

### 4.15. DAF-16::GFP Localization

The *C. elegans* strain TJ356 was cultured for 2 days in NGM supplemented with apiin until it reached the young adult stage, and then harvested by washing three times with M9 buffer. The worms were then sedated using 25 mM levamisole and placed on 2% agarose pads for fluorescence imaging with a 10 × objective lens. The localization of GFP was categorized into cytoplasmic, intermediate, and nuclear types. The cytoplasmic state is characterized by uniform fluorescence distributed throughout the cytoplasm. The intermediate state is defined by diffuse cytoplasmic fluorescence accompanied by bright nuclear fluorescence in specific regions of the worm body, such as the anterior and posterior regions. The nuclear state is marked by significant fluorescence enrichment within the cell nucleus, which is clearly visible in multiple nuclei throughout the worm body. This classification system is based on prior research and determines the degree of translocation from the cytoplasm to the nucleus by analyzing DAF-16::GFP expression patterns [65,66]. In each replicate, approximately 20 *C. elegans* worms were analysed, and three biological replicates were maintained.

### 4.16. Real-Time Quantitative PCR (RT-qPCR)

After a 10-day treatment with apiin, total RNA was extracted from N2 *C. elegans* using the RNAeasy™ Animal RNA Isolation Kit from Beyotime (China). For cDNA synthesis, the HiScript III 1st Strand cDNA Synthesis Kit from Vazyme (Nanjing, China) was used. RT-qPCR was performed using the ArtiCanATM SYBR qPCR Mix from Tsingke (Beijing, China), following the manufacturer’s instructions. Gene expression was evaluated using the 2^−ΔΔCt^ method, with act-1 serving as the internal control for normalization purposes. The primers utilized are detailed in Table S1.

### 4.17. TG Assay

In total, 2000 N2 *C. elegans* individuals from each group were collected and wash three times with M9, and the supernatant was discarded. Afterwards, 100–200 µL of PBS containing 1% protease inhibitor was added. The sample was frozen promptly in liquid nitrogen and then thawed in a water bath at 37 °C. Three freeze–thaw cycles were conducted to help break down the cuticle. Further disruption was performed on ice using an ultrasonic disruptor or a glass homogenizer. The mixture was subsequently centrifuged at 12,000× *g* for 10 min at 4 °C, after which the supernatant was collected or further analysis. The enzyme working solution was prepared according to the given protocols. The solution was incubated for 15 to 30 min at 37 °C in the dark. The absorbance (OD) was recorded at 540 nm (or at the wavelength specified in the kit’s instructions). The concentration of TG in the sample was calculated using the formula: Sample TG concentration = (Standard OD − Blank OD)/(Sample OD − Blank OD) multiplied by the standard concentration and adjusted for dilution.

### 4.18. RNA Sequencing

Adult N2 *C. elegans* were placed on an NGM plate containing 100 μg/mL apiin. Following the spawning period, they were simultaneously reared at 20 °C until they developed into Day 5 of adulthood specimens. To remove the OP50, the worms were rinsed with M9 buffer, collected, and subsequently flash-frozen in liquid nitrogen for storage at −80 °C. These samples were then sent to BGI Genomics Co., Ltd. (Wuhan, China) for further library construction analysis. *C. elegans* worm samples from various treatment groups were collected for transcriptional sequencing. Following quality assessment, the gene expression levels were quantitatively analysed through principal component analysis, correlation assessments, differential gene screening, etc. Differentially expressed genes identified among the samples were subjected to further comprehensive analyses including GO functional significance enrichment analysis, KEGG significance enrichment analysis, clustering, protein interaction network analysis, and transcription factor analysis. The detailed methodology can be found in the Supplementary Materials.

### 4.19. Metabolomic Profile

Cultures and N2 *C. elegans* underwent identical treatments as those used for RNA sequencing. The collected *C. elegans* samples were subsequently sent to BGI Genomics Co., Ltd. (Shenzhen, China) for further analysis of differential metabolites. The experimental procedure consisted of metabolite extraction, UPLC-MS analysis, and subsequent software evaluation. A Q Exactive HF mass spectrometer (Thermo Fisher Scientific, Waltham, MA, USA) was used for the acquisition of both primary and secondary mass spectrometry data. The resulting mass spectrometry data were imported into Compound Discoverer 3.3 software (Thermo Fisher Scientific, USA) and analysed alongside the BGI Metabolome Database (BMDB), mzCloud database, and ChemSpider online resources. Then, a data matrix containing information on the peak areas of the metabolites and the corresponding identification results was produced. This table was subsequently subjected to further analysis and processing to extract relevant information. The detailed methods can be found in the Supplementary Materials.

### 4.20. Statistical Analysis

GraphPad Prism 8.01 software was used to create the graphs and perform the statistical analyses. The experiments were conducted in triplicate, and the results are presented as the mean ± SD. To assess significance across multiple groups, one-way ANOVA was employed, followed by Tukey’s test for multiple comparisons; a *p* value < 0.05 indicated a significant difference, whereas a *p* value < 0.01 indicated a highly significant difference. The log-rank test was used to evaluate the survival curves of *C. elegans* for statistical significance.

## 5. Conclusions

Apiin is a natural antioxidant. This flavonoid enhances lipid metabolism and antioxidant capacity by regulating DAF-16, thereby achieving anti-aging effects. It holds promise for in-depth research as a nutritional supplement. Simultaneously, we should deepen our exploration of plant-derived natural compounds to discover more bioactive ingredients beneficial to health.

## Data Availability

The original contributions presented in this study are included in the article/Supplementary Material. Further inquiries can be directed to the corresponding authors.

## Supplementary Materials

The following supporting information can be downloaded at https://www.mdpi.com/article/10.3390/ijms262411888/s1. Reference [67] is cited in the Supplementary Materials.

## Abbreviations

The following abbreviations are used in this manuscript:

*C. elegans*	*Caenorhabditis elegans*
ROS	Reactive oxygen species
TSPJ	Total saponins from Panax japonicus
AGEs	Advanced glycation end products
PVAT	Perivascular adipose tissue
NR	Nile red
ORO	Oil red O
RT-qPCR	Real-time quantitative PCR
TG	Triglyceride
