# Supplementary material for: Apiin Promotes Healthy Aging in C. elegans Through Nutritional Activation of DAF-16/FOXO, Enhancing Fatty Acid Catabolism and Oxidative Stress Resistance
Source: Int J Mol Sci. 2025 Dec 10;26(24):11888. doi: 10.3390/ijms262411888 (PMC12732714; doi:10.3390/ijms262411888)
Supplement: Supplementary file 1 [file ijms-26-11888-s001.zip › ijms-3962881-supplementary.pdf]

Apiin Promotes Healthy Aging in *C. elegans* by Nutritional Activation of DAF-16/FOXO, Enhancing Fatty Acid Catabolism and Oxidative Stress Resistance

To whom Correspondence should be addressed:

five841110@126.com

Table of contents

Figure S1. Apiin's quality control report.

Table S1. List of primers used for the quantitative real-time reverse transcription-polymerase chain reaction.

Table S2. Related genes and connection numbers of KEGG pathway genes.

Figure S2 Association network diagram.

Other supplementary materials:

RNA-seq Materials and methods

Metabolomics Materials and methods

Figure S1. Apiin's quality control report.

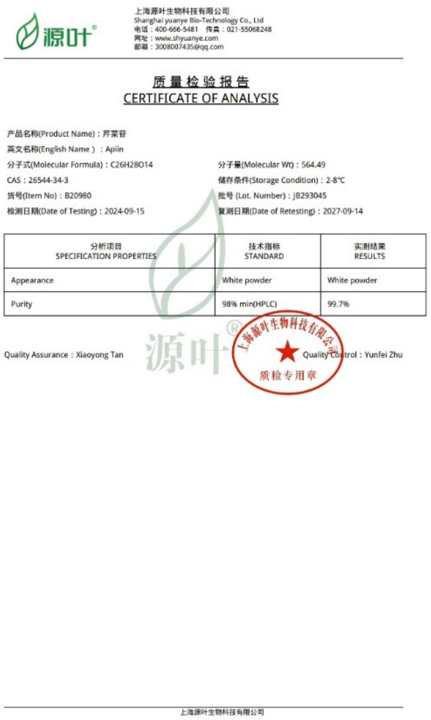

上海源叶生物科技有限公司
Shanghai yuanye Bio-Technology Co., Ltd
电话：400-666-5481 传真：021-55068248
网址：www.shyuanye.com
邮箱：3008007435@qq.com

质量检验报告
CERTIFICATE OF ANALYSIS

产品名称(Product Name)：芹菜苷
英文名称(English Name）：Apiin
分子式(Molecular Formula)：C26H28O14
分子量(Molecular Wt)：564.49
CAS：26544-34-3
储存条件(Storage Condition)：2-8℃
货号(Item No)：B20980
批号 (Lot. Number)：JB293045
检测日期(Date of Testing)：2024-09-15
复测日期(Date of Retesting)：2027-09-14

分析项目 SPECIFICATION PROPERTIES	技术指标 STANDARD	实测结果 RESULTS
Appearance	White powder	White powder
Purity	98% min(HPLC)	99.7%

Quality Assurance：Xiaoyong Tan   Quality Control：Yunfei Zhu

上海源叶生物科技有限公司 质检专用章

上海源叶生物科技有限公司

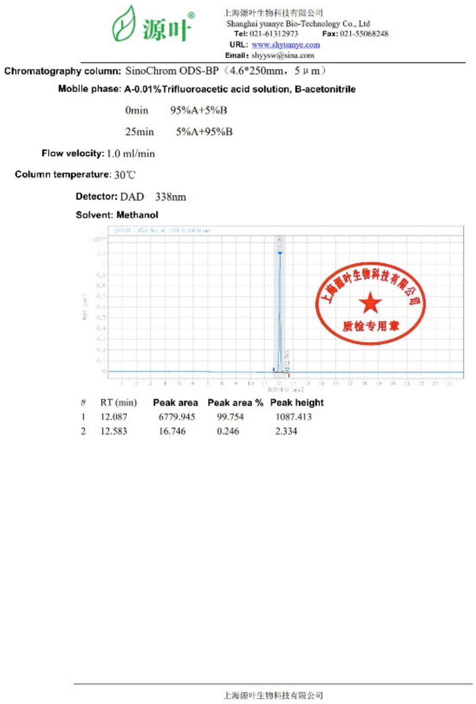

上海源叶生物科技有限公司
Shanghai yuanye Bio-Technology Co., Ltd
Tel: 021-61312973 Fax: 021-55068248
URL: www.shyuanye.com
Email: shyysw@sina.com

Chromatography column: SinoChrom ODS-BP（4.6*250mm，5 μm）
Mobile phase: A-0.01%Trifluoroacetic acid solution, B-acetonitrile
0min 95%A+5%B
25min 5%A+95%B
Flow velocity: 1.0 ml/min
Column temperature: 30℃
Detector: DAD 338nm
Solvent: Methanol

#	RT (min)	Peak area	Peak area %	Peak height
1	12.087	6779.945	99.754	1087.413
2	12.583	16.746	0.246	2.334

上海源叶生物科技有限公司 质检专用章

上海源叶生物科技有限公司

Table S1. List of primers used for the quantitative real-time reverse transcription-polymerase chain reaction

Gene name	Type	primer sequence(5' to 3')
Daf-16	F	CTAACTTCAAGCCAATGCCACTA
Daf-16	R	TCCAGCTTGACTCAGCTCATGTC
Daf-2	F	TGGATCTCCATCGCGAAACG
Daf-2	R	TTTTGGGGGTTTCAGACAAGT
Hsp-12.6	F	CGTCGAGGACCTGGAATCAA
Hsp-12.6	R	GATGGCTGACGAAGGAACCA
Sod-3	F	TGGGAAGTTCATGTTGAACGAG
Sod-3	R	AGGATCCTGGTTTGCACAGG
Mtl-1	F	TTTCTCACTGGCCTCCTCAC
Mtl-1	R	TCATGGCTTGCAAGTGTGGT
Ech-9	F	CCCATAGTTCCACCGCCAAT
Ech-9	R	GCGGGACAGGTGATTTCATTT
Act-1	F	TGCCCCATCGTAAGTTTTCT
Act-1	R	ACTTGCGGTGAACGATGGAT

Table S2 Related genes and connection numbers of KEGG pathway genes

Gene ID	Gene Symbol	Type	ppi_total	ppi_173776	ppi_187683	ppi_185299	ppi_183367	ppi_179060	ppi_181748	ppi_177778	ppi_184065
172981	'daf-16'	mRNA	4	1	0	0	0	1	1	1	0
259738	'ctl-1'	mRNA	4	1	0	0	0	1	1	1	0
173078	'hsf-1'	mRNA	3	0	0	0	0	1	1	1	0
175085	'ctl-2'	mRNA	3	1	0	0	0	1	1	0	0
175086	'ctl-3'	mRNA	3	1	0	0	0	1	1	0	0
175410	'daf-2'	mRNA	3	0	0	0	0	1	1	1	0
177343	'skn-1'	mRNA	3	0	0	0	0	1	1	1	0
177886	'gst-4'	mRNA	3	0	0	1	0	1	1	0	0
178659	'hsp-16.2'	mRNA	3	0	0	0	0	1	1	1	0
181748	'sod-3'	mRNA	3	1	0	0	0	1	0	1	0
187542	'gpx-7'	mRNA	3	1	0	1	0	0	1	0	0
171951	'C32E8.9'	mRNA	2	0	0	1	0	0	0	0	1
172195	'atp-3'	mRNA	2	1	0	0	0	0	1	0	0
172632	'sod-2'	mRNA	2	1	0	0	0	0	1	0	0
173776	'sod-5'	mRNA	2	0	0	0	0	1	1	0	0
174141	'sod-1'	mRNA	2	1	0	0	0	0	1	0	0
174438	'gcs-1'	mRNA	2	1	0	0	0	0	1	0	0
174762	'age-1'	mRNA	2	0	0	0	0	1	1	0	0
175467	'gsr-1'	mRNA	2	1	0	1	0	0	0	0	0
175766	'hach-1'	mRNA	2	0	0	0	0	0	1	0	1
176102	'cuc-1'	mRNA	2	1	0	0	0	1	0	0	0
176336	'sod-4'	mRNA	2	1	0	0	0	0	1	0	0
176471	'sip-1'	mRNA	2	0	0	0	0	1	0	1	0
176837	'prdx-6'	mRNA	2	1	0	1	0	0	0	0	0
177778	'hsp-12.6'	mRNA	2	0	0	0	0	1	1	0	0
178016	'ard-1'	mRNA	2	1	0	0	0	0	0	0	1
179060	'mtl-1'	mRNA	2	0	0	0	0	0	1	1	0
179289	'hsp-16.11'	mRNA	2	0	0	0	0	1	0	1	0
179424	'akt-1'	mRNA	2	1	0	0	0	0	1	0	0
179627	'sodh-1'	mRNA	2	0	0	1	0	1	0	0	0
181178	'gpx-5'	mRNA	2	1	0	1	0	0	0	0	0
181524	'akt-2'	mRNA	2	1	0	0	0	0	1	0	0
184981	'gpx-1'	mRNA	2	1	0	1	0	0	0	0	0
188313	'gpx-6'	mRNA	2	1	0	0	0	0	1	0	0
1.3E+07	'C02D5.4'	mRNA	1	0	0	1	0	0	0	0	0
1.3E+07	'Y55F3BL.4'	mRNA	1	0	0	0	0	0	1	0	0
171619	'C53D5.5'	mRNA	1	0	0	1	0	0	0	0	0
171710	'mrpl-15'	mRNA	1	0	0	0	0	0	1	0	0
171889	'mrpl-17'	mRNA	1	0	0	0	0	0	1	0	0
172052	'mrpl-19'	mRNA	1	0	0	0	0	0	1	0	0
172301	'T09B4.8'	mRNA	1	0	0	0	0	0	0	0	1
172310	'ech-1.2'	mRNA	1	0	0	0	0	0	0	0	1
172438	'nuo-6'	mRNA	1	1	0	0	0	0	0	0	0
172479	'mrpl-13'	mRNA	1	0	0	0	0	0	1	0	0
172535	'F27D4.1'	mRNA	1	1	0	0	0	0	0	0	0
172538	'F27D4.4'	mRNA	1	0	1	0	0	0	0	0	0
172674	'pbs-7'	mRNA	1	1	0	0	0	0	0	0	0
172757	'hsp-70'	mRNA	1	0	0	0	0	0	0	1	0
172850	'mekk-3'	mRNA	1	0	0	0	1	0	0	0	0
172939	'ndk-1'	mRNA	1	1	0	0	0	0	0	0	0
172942	'pas-5'	mRNA	1	1	0	0	0	0	0	0	0
172983	'gpx-8'	mRNA	1	1	0	0	0	0	0	0	0
173162	'acox-1.1'	mRNA	1	0	0	0	0	0	0	0	1
173163	'acox-1.2'	mRNA	1	0	0	0	0	0	0	0	1
173164	'acox-1.4'	mRNA	1	0	0	0	0	0	0	0	1
173220	'Y48G10A.1'	mRNA	1	1	0	0	0	0	0	0	0
173300	'ech-7'	mRNA	1	0	0	0	0	0	0	0	1
173416	'djr-1.1'	mRNA	1	1	0	0	0	0	0	0	0
173466	'acdh-9'	mRNA	1	0	0	0	0	0	0	0	1

Figure S2 Association network diagram

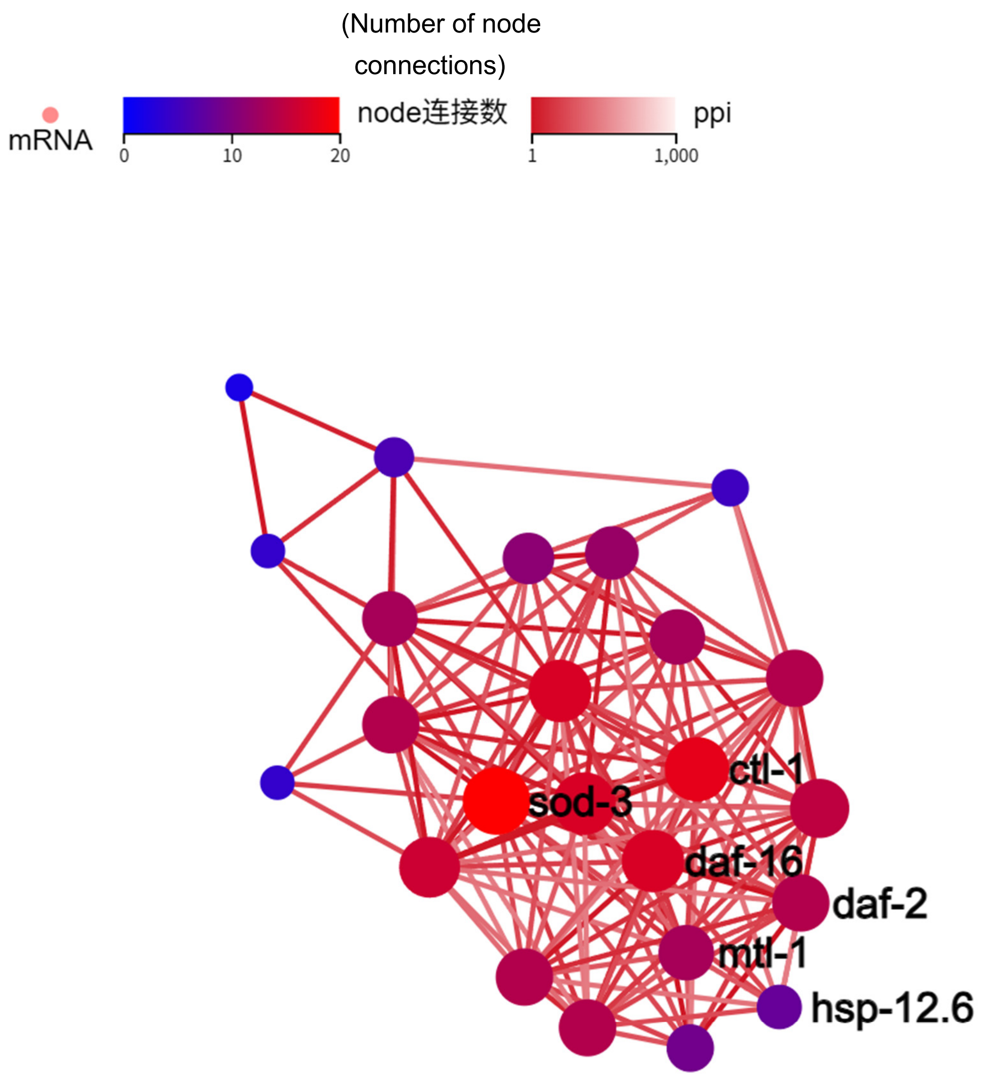

RNA-seq Materials and methods

Experimental procedure

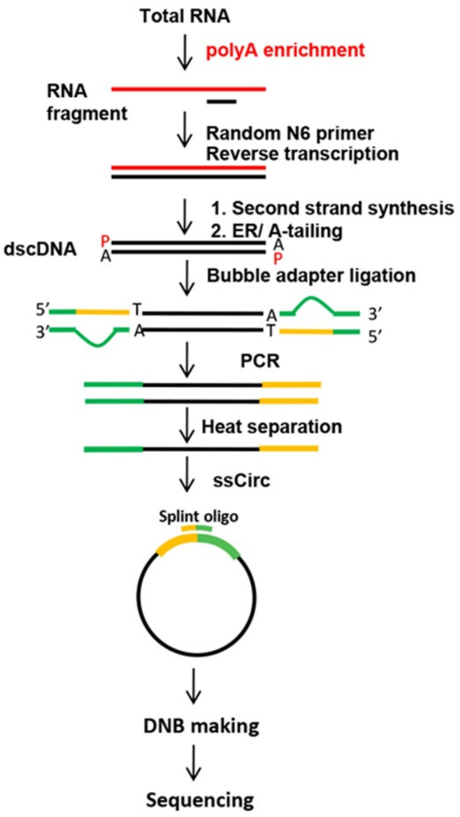

1)mRNA enrichment and purification: Oligo dT Selection to enrich the mRNA (For total RNA extracted from human whole blood, globin mRNA are depleted); 2)RNA fragmentation and cDNA synthesis (second-strand cDNA synthesis with dUTP instead of dTTP); 3)End repair, add A and adaptor ligation; 4)PCR; 5)Circularization and make DNB; 6) Sequencing on DNBSEQ platform

Analysis Pipline

Sequencing data is called raw reads or raw data, and quality control (QC) is then performed on the raw reads to determine whether the sequencing data is suitable for subsequent analysis. After quality control, the filtered clean reads were aligned to the reference sequence. After the alignment, the statistics of the mapping rate and the distribution of reads on the reference sequence are used to determine whether the alignment result passes the second QC of alignment. If it passes, we perform gene quantification analysis and other analysis based on gene expression (principal component, correlation, differential gene screening, etc.), and perform significant enrichment analysis of GO function on differentially expressed genes among the screened samples, significance enrichment analysis of pathway, clustering, protein interaction networks, and transcription factors, and more in-depth mining analysis. SNP & InDel, differential splicing gene detection can also be done. The complete analysis flow chart is as follows:

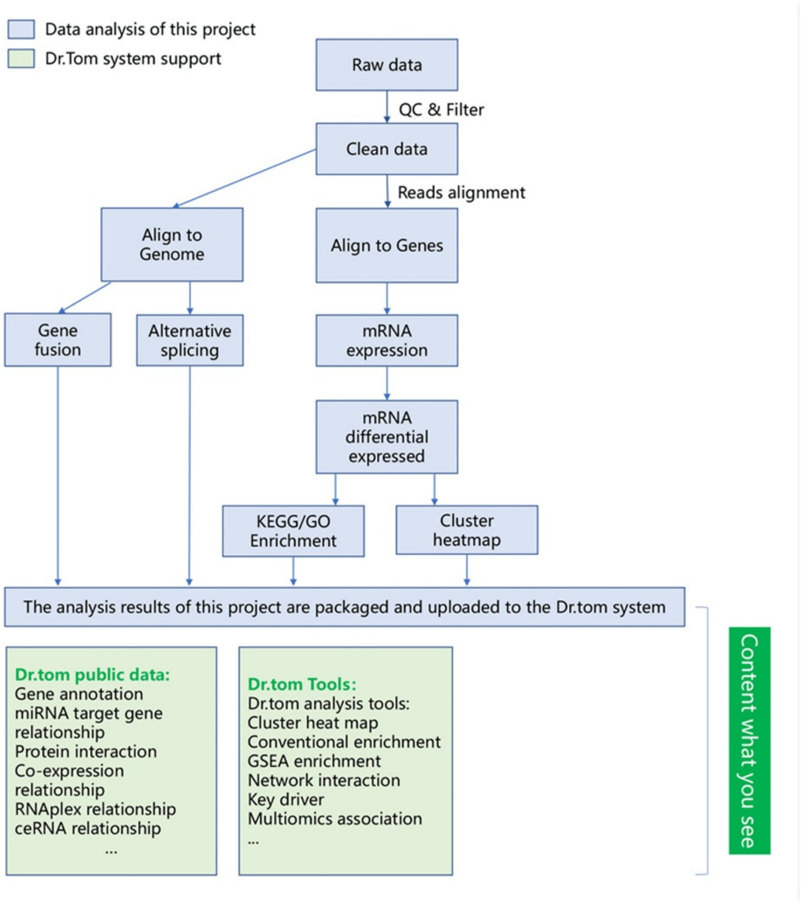

Sequencing data filtering

This project uses the filtering software SOAPnuke developed by BGI independently for filtering. The specific steps are as follows:
1) Remove the reads containing the adaptor(adaptor pollution);
2) Remove reads whose N content is greater than 1%;
3) Remove low-quality reads (we define reads with bases with a quality score no more than 20 as the proportion of total bases in the reads that are greater than 40% as low-quality reads).
The filtered "Clean Reads" are saved in FASTQ format.

Software Information
SOAPnuke:
Version v2.3
Parameters -l 20 -q 0.4 -n 0.01
Official Websitehttps://github.com/BGI-flexlab/SOAPnuke
References
[1]Cock P., et al.(2010). The Sanger FASTQ file format for sequences with quality scores, and the Solexa/Illumina FASTQ variants. Nucleic Acids Research, 38(6): 1767-1771.

Use Bowtie2 (v2.3.4.3) to contrast clean data to reference gene sets. Quantify gene expression using RSEM (v1.3.1) software, and draw heatmaps of gene expression clustering in different samples using pheatmap (v1.0.8). Differential gene detection was performed using DESeq2 (v1.4.5) (or DEGseq or PoissonDis), provided that the P value was ≤0.05 or |log2FC|≥1.2.

Metabolomics Materials and methods

Metabolite overall analysis could provide the metabolites both qualitative and quantitative description. Qualitatively, it displays the detection effect on chromatogram from different groups, and also shows the classification of the identified metabolites and the number statistics of each metabolite. Quantitatively, the similarity of each comparison group was observed through the PCA plot, and the expression patterns among the groups were displayed through clustering.

Chromatogram

Animal,Plant,Lipidomics: Under both positive and negative mode ,one sample from each group was selected for the chromatographic display of base peak ion (BPC). It could be seen from the BPC diagram that the detection peak shape of the samples was good and the peak capacity was large. Widely targeted,HM400: The typical extracted ion current diagram (XIC) of the sample can visually show the detection of metabolites in the sample.

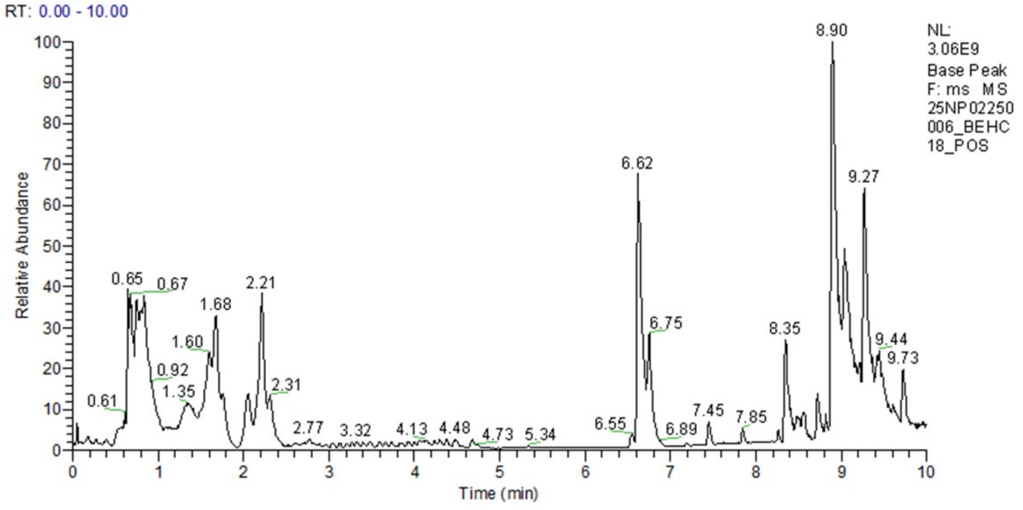

Differential screening

An univariate analysis used Fold change (FC) and p-value to select Differential Metabolite Screening, and map a Volcano Plot. A multivariate statistical Analysis method such as PCA (Principal Component Analysis) could state the over all difference between groups. Differential metabolites screening criteria: 1) $|log2FC| \geq 1.2$, 2) p-value < 0.05.

PCA

A PCA model was established between the comparative analysis group to observe the distribution and separation trend of the two groups of samples. The PCA figure here shows how two principal components can reveal the difference between sample groups, and separation trend in each sample group, while keeping the original variable information.

Content analysis-heat map

Cluster analysis of the expression levels of the differential metabolites from a comparison group can intuitively see the expression rules of the differential metabolites in the two groups of samples. The data were log2 transformed and z-score normalized during analysis, The clustering algorithm uses Hierarchical Cluster, and the distance calculation uses Euclidean distance.

KEGG enrichment analysis

Metabolic pathway enrichment analysis of differential metabolites based on the KEGG database can reveal significantly altered metabolic pathways, thereby contributing to the interpretation of biological phenotypes. In this report, the metabolic pathways with *p* value<0.05 were defined as the metabolic pathways with significant enrichment of differential metabolite.

ADMETlab 3.0

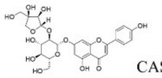

Apiin
CAS： 26544-34-3

C@@H]3O[C@H](CO)[C@@H](O)[C@H](O)[C@H]3O[C@@H]3OC[C@](O)(CO)[C@H]3O)cc(O)c12

1. Physicochemical Property

Property	Value	Comment
Molecular Weight	564.15	Contain hydrogen atoms. Optimal:100~600
Volume	517.441	Van der Waals volume
Density	1.09	Density = MW / Volume
nHA	14.0	Number of hydrogen bond acceptors. Optimal:0~12
nHD	8.0	Number of hydrogen bond donors. Optimal:0~7
nRot	7.0	Number of rotatable bonds. Optimal:0~11
nRing	5.0	Number of rings. Optimal:0~6
MaxRing	10.0	Number of atoms in the biggest ring. Optimal:0~18
nHet	14.0	Number of heteroatoms. Optimal:1~15
fChar	0.0	Formal charge. Optimal:-4 ~4
nRig	29.0	Number of rigid bonds. Optimal:0~30
Flexibility	0.241	Flexibility = nRot /nRig
Stereo Centers	8.0	Stereo Centers. Optimal: ≤ 2
TPSA	228.97	Topological Polar Surface Area. Optimal:0~140
logS	-3.495	The logarithm of aqueous solubility value.
logP	0.822	The logarithm of the n-octanol/water distribution coefficients at pH=7.4.
logD	1.014	The logarithm of the n-octanol/water distribution coefficient.
pka (Acid)	7.146	Acid-base dissociation constant (pKa) value represents the strength of a drug molecule's acidity or basicity.
pka (Base)	4.435	Acid-base dissociation constant (pKa) value represents the strength of a drug molecule's acidity or basicity.
Melting point	261.91	The predicted melting point of a compound is expressed in degrees Celsius (°C).<br>Melting points below 25°C are classified as liquids, while melting points above 25°C are classified as solids.
Boiling point	376.298	The predicted melting point of a compound is expressed in degrees Celsius (°C).<br>A normal boiling point below 25°C is categorized as a gas.

2. Medicinal Chemistry

Property	Value	Decision	Comment
QED	0.164	●	■ A measure of drug-likeness based on the concept of desirability;<br>■ Attractive: > 0.67;<br>■ unattractive: 0.49~0.67;<br>■ too complex: < 0.34
GASA	1.0	●	■ ES: Easy to synthesize; HS: Hard to synthesize;<br>■ The output value represents the probability of being difficult to synthesize, ranging from 0 to 1.
Synth	4.0	●	■ Synthetic accessibility score is designed to estimate ease of synthesis of drug-like molecules.<br>■ SAscore ≥ 6, difficult to synthesize; SAscore <6, easy to synthesize
Fsp3	0.423	●	■ The number of sp3 hybridized carbons / total carbon count, correlating with melting point and solubility.<br>■ Fsp^3 ≥0.42 is considered a suitable value.
MCE-18	115.054	●	■ MCE-18 stands for medicinal chemistry evolution.<br>■ MCE-18≥45 is considered a suitable value.
NPscore	2.259	-	■ Natural product-likeness score.<br>■ This score is typically in the range from -5 to 5.<br>■ The higher the score is, the higher the probability is that the molecule is a NP.
Lipinski Rule	1.0	●	■ MW ≤ 500; logP ≤ 5; Hacc ≤ 10; Hdon ≤ 5<br>■ If two properties are out of range, a poor absorption or permeability is possible, one is acceptable.
Pfizer Rule	0.0	●	■ logP > 3; TPSA < 75<br>■ Compounds with a high log P (>3) and low TPSA (<75) are likely to be toxic.
GSK Rule	1.0	●	■ MW ≤ 400; logP ≤ 4<br>■ Compounds satisfying the GSK rule may have a more favorable ADMET profile
Golden Triangle	1.0	●	■ 200 ≤ MW ≤ 500; -2 ≤ logD ≤ 5<br>■ Compounds satisfying the Golden Triangle rule may have a more favorable ADMET profile.
PAINS	0 alerts	-	frequent hitters, Alpha-screen artifacts and reactive compound 480 substructures (J Med Chem 201053:2719-40)
ALARM NMR	2 alerts	-	Thiol reactive compounds.
BMS	0 alerts	-	undesirable, reactive compounds 176 substructures (J Chem Inf Model 200646:1060-8)
Chelator Rule	0 alerts	-	Chelating compounds.
Colloidal aggregators	0.611	-	■ Category 0: non-colloidal aggregators;<br>■ Category 1: colloidal aggregators.<br>■ The output value is the probability of being colloidal aggregators, within the range of 0 to 1.

			inhibitors, within the range of 0 to 1.
Blue fluorescence	0.951	●	■ Category 0: non-blue fluorescence;<br>■ Category 1: blue fluorescence.<br>■ The output value is the probability of being blue fluorescence, within the range of 0 to 1.
Green fluorescence	0.556	●	■ Category 0: non-green fluorescence;<br>■ Category 1: green fluorescence.<br>■ The output value is the probability of being green fluorescence, within the range of 0 to 1.
Reactive compounds	0.183	●	■ Category 0: non-reactive compound;<br>■ Category 1: reactive compound.<br>■ The output value is the probability of being reactive compound, within the range of 0 to 1.
Promiscuous compounds	0.51	●	■ Category 0: non-promiscuous compound;<br>■ Category 1: promiscuous compound.<br>■ The output value is the probability of being promiscuous compound, within the range of 0 to 1.

3. Absorption

Property	Value	Decision	Comment
Caco-2 Permeability	-6.538	●	Optimal: higher than -5.15 Log unit
MDCK Permeability	-5.054	●	■ low permeability: < 2 × 10-6 cm/s<br>■ medium permeability: 2-20 × 10^{-6} cm/s<br>■ high passive permeability: > 20 × 10^{-6} cm/s
PAMPA	0.999	●	■ The experimental data for Peff was logarithmically transformed (logPeff).<br>■ Molecules with log Peff values below 2.0 were classified as low-permeability (Category 0), while those with log Peff values exceeding 2.5 were classified as high-permeability (Category 1).
Pgp-inhibitor	0.0	●	■ Category 1: Inhibitor;<br>■ Category 0: Non-inhibitor;<br>■ The output value is the probability of being Pgp-inhibitor
Pgp-substrate	0.576	●	■ Category 1: substrate;<br>■ Category 0: Non-substrate;<br>■ The output value is the probability of being Pgp-substrate
HIA	0.896	●	■ Human Intestinal Absorption<br>■ Category 1: HIA+(HIA < 30%);<br>■ Category 0: HIA-(HIA >= 30%);<br>■ The output value is the probability of being HIA+

%	0.896	●	■ 20% Bioavailability<br>■ Category 1: F 20% + (bioavailability < 20%);<br>■ Category 0: F 20% - (bioavailability ≥ 20%);<br>■ The output value is the probability of being F 20% +
$F_{30\%}$	1.0	●	■ 30% Bioavailability<br>■ Category 1: F 30% + (bioavailability < 30%);<br>■ Category 0: F 30% - (bioavailability ≥ 30%);<br>■ The output value is the probability of being F 30% +
$F_{50\%}$	1.0	●	■ 50% Bioavailability<br>■ Category 1: F 50% + (bioavailability < 50%);<br>■ Category 0: F 50% - (bioavailability ≥ 50%);<br>■ The output value is the probability of being F 50% +

4. Distribution

Property	Value	Decision	Comment
PPB	80.867	●	■ Plasma Protein Binding<br>Optimal: < 90%.<br>■ Drugs with high protein-bound may have a low therapeutic index.
VDss	-0.043	●	■ Volume Distribution<br>■ Optimal: 0.04-20L/kg
BBB	0.003	●	■ Blood-Brain Barrier Penetration<br>■ Category 1: BBB+; Category 0: BBB-;<br>■ The output value is the probability of being BBB+
Fu	18.264	●	■ The fraction unbound in plasms<br>■ Low: <5%; Middle: 5~20%; High: > 20%
OATP1B1 inhibitor	0.999	●	■ Category 0: Non-inhibitor; Category 1: inhibitor.<br>■ The output value is the probability of being inhibitor, within the range of 0 to 1.
OATP1B3 inhibitor	1.0	●	■ Category 0: Non-inhibitor; Category 1: inhibitor.<br>■ The output value is the probability of being inhibitor, within the range of 0 to 1.
BCRP inhibitor	0.23	●	■ Category 0: Non-inhibitor; Category 1: inhibitor.<br>■ The output value is the probability of being inhibitor, within the range of 0 to 1.
MRP1 inhibitor	0.471	●	■ Category 0: Non-inhibitor; Category 1: inhibitor.<br>■ The output value is the probability of being inhibitor, within the range of 0 to 1.

5. Metabolism

Property	Value	Decision	Comment
CYP1A2 inhibitor	0.056	●	■ Category 1: Inhibitor; Category 0: Non-inhibitor;<br>■ The output value is the probability of being inhibitor.

CYP1A2 substrate	0.0	🟢	■ Category 1: Substrate; Category 0: Non-substrate; ■ The output value is the probability of being substrate.
CYP2C19 inhibitor	0.0	🟢	■ Category 1: Inhibitor; Category 0: Non-inhibitor; ■ The output value is the probability of being inhibitor.
CYP2C19 substrate	0.0	🟢	■ Category 1: Substrate; Category 0: Non-substrate; ■ The output value is the probability of being substrate.
CYP2C9 inhibitor	0.0	🟢	■ Category 1: Inhibitor; Category 0: Non-inhibitor; ■ The output value is the probability of being inhibitor.
CYP2C9 substrate	0.001	🟢	■ Category 1: Substrate; Category 0: Non-substrate; ■ The output value is the probability of being substrate.
CYP2D6 inhibitor	0.0	🟢	■ Category 1: Inhibitor; Category 0: Non-inhibitor; ■ The output value is the probability of being inhibitor.
CYP2D6 substrate	0.001	🟢	■ Category 1: Substrate; Category 0: Non-substrate; ■ The output value is the probability of being substrate.
CYP3A4 inhibitor	0.118	🟢	■ Category 1: Inhibitor; Category 0: Non-inhibitor; ■ The output value is the probability of being inhibitor.
CYP3A4 substrate	0.0	🟢	■ Category 1: Substrate; Category 0: Non-substrate; ■ The output value is the probability of being substrate.
CYP2B6 inhibitor	0.002	🟢	■ Category 1: Inhibitor; Category 0: Non-inhibitor; ■ The output value is the probability of being inhibitor.
CYP2B6 substrate	0.0	🟢	■ Category 1: Substrate; Category 0: Non-substrate; ■ The output value is the probability of being substrate.
CYP2C8 inhibitor	0.295	🟢	■ Category 1: Inhibitor; Category 0: Non-inhibitor; ■ The output value is the probability of being inhibitor.
HLM Stability	0.021	🟢	■ human liver microsomal (HLM) stability ■ Category 0: stable+ (HLM > 30 min); Category 1: unstable- (HLM ≤ 30 min). The output value is the probability of human liver microsomal instability, where a value closer to 1 indicates a higher likelihood of instability. The range is between 0 and 1.

6. Excretion

Property	Value	Decision	Comment
CL_{plasma}	2.063	🟢	■ The unit of predicted CLplasma penetration is ml/min/kg. >15 ml/min/kg: high clearance; 5-15 ml/min/kg: moderate clearance; < 5 ml/min/kg: low clearance.
$T_{1/2}$	3.049	🟡	■ The unit of predicted T1/2 is hours. ■ ultra-short half-life drugs: 1/2 < 1 hour; short half-life drugs: T1/2 between 1-4 hours; intermediate short half-life drugs: T1/2 between 4-8 hours; long half-life drugs: T1/2 > 8 hours.

7. Toxicity

Property	Value	Decision	Comment
hERG Blockers	0.017	🟢	■ Molecules with IC50 ≤10µM or ≥50% inhibition at 10 µM were classified as hERG+ (Category 1), ■ while molecules with IC50 >10µM or < 50% inhibition at 10µM were classified as hERG - (Category 0). ■ The output value is the probability of being hERG+, within the range of 0 to 1.
hERG Blockers (10um)	0.055	🟢	■ Molecules with IC50 ≤10 µM are classified as hERG+ (Category 1), ■ and molecules with IC50 > 10µM are classified as hERG- (Category 0). ■ The output value is the probability of being hERG+, within the range of 0 to 1.
DILI	0.978	🔴	■ Drug Induced Liver Injury. ■ Category 1: drugs with a high risk of DILI; ■ Category 0: drugs with no risk of DILI. ■ The output value is the probability of being toxic.
AMES Muta genicity	0.955	🔴	■ AMES Toxicity ■ Category 1: Ames positive(+); ■ Category 0: Ames negative(-); ■ The output value is the probability of being toxic.
Rat Oral Acute Toxicity	0.046	🟢	■ Rat Oral Acute Toxicity. ■ Category 0: low-toxicity, > 500 mg/kg; ■ Category 1: high-toxicity; < 500 mg/kg. ■ The output value is the probability of being toxic, within the range of 0 to 1.
FDAMDD	0.094	🟢	■ FDA Maximum (Recommended) Daily Dose. ■ Category 1: FDAMDD (+); ■ Category 0: FDAMDD (-); The output value is the probability of being positive.
Skin Sensiti zation	1.0	🔴	■ Category 1: Sensitizer; ■ Category 0: Non-sensitizer. ■ The output value is the probability of being toxic, within the range of 0 to 1.
Carcinogeni city	0.653	🟠	■ Category 1: carcinogens; ■ Category 0: non-carcinogens; ■ The output value is the probability of being toxic.

Eye Corrosion	0.0	🟢	■ Eye Corrosion ■ Category 1: corrosives; Category 0: noncorrosives; ■ The output value is the probability of being corrosives.
Eye Irritation	0.562	🟠	■ Eye Irritation ■ Category 1: irritants; Category 0: nonirritants; ■ The output value is the probability of being irritants.
Respiratory	0.034	🟢	■ Category 1: respiratory toxicants; ■ Category 0: non-respiratory toxicants. ■ The output value is the probability of being toxic, within the range of 0 to 1.
Human Hep atotoxicity	0.787	🔴	■ Human Hepatotoxicity ■ Category 1: H-HT positive(+); ■ Category 0: H-HT negative(-); ■ The output value is the probability of being toxic.
Drug-induce d Nephrotox icity	0.424	🟠	■ Category 0: non-nephrotoxic (-); ■ Category 1: nephrotoxic (+). ■ The output value is the probability of being nephrotoxic (+), within the range of 0 to 1.
Ototoxicity	0.862	🔴	■ Category 0: non-ototoxicity (-); ■ Category 1: ototoxicity (+). ■ The output value is the probability of being ototoxicity (+), within the range of 0 to 1.
Hematotoxic ity	0.112	🟢	■ Category 0: non-hematotoxicity (-); ■ Category 1: hematotoxicity (+). ■ The output value is the probability of being hematotoxicity (+), within the range of 0 to 1.
Genotoxicity	0.989	🔴	■ Category 0: non-Genotoxicity (-); ■ Category 1: Genotoxicity (+). ■ The output value is the probability of being ototoxicity (+), within the range of 0 to 1.
RPMI-8226 Immunitoxici ty	0.132	🟢	■ Category 0: non-cytotoxicity (-); ■ Category 1: cytotoxicity (+). ■ The output value is the probability of being ototoxicity (+), within the range of 0 to 1.
A549 Cytotoxicity	0.698	🟠	■ Category 0: non-cytotoxicity (-); ■ Category 1: cytotoxicity (+). ■ The output value is the probability of being ototoxicity (+), within the range of 0 to 1.
Hek293 Cytotoxicity	0.828	🔴	■ Category 0: non-cytotoxicity (-); ■ Category 1: cytotoxicity (+). ■ The output value is the probability of being ototoxicity (+), within the range of 0 to 1.
Drug-induce d Neurotoxi city	0.005	🟢	■ Category 0: non-neurotoxic (-); ■ Category 1: neurotoxic (+). ■ The output value is the probability of being neurotoxic (+), within the range of 0 to 1.

8. Environmental toxicity

Property	Value	Comment
Bioconcentration Factors	0.345	■ Bioconcentration factors are used for considering secondary poisoning potential and assessing risks to human health via the food chain. ■ The unit is –log10[(mg/L)/(1000*MW)]
IGC_{50}	2.775	■ Tetrahymena pyriformis 50 percent growth inhibition concentration. ■ The unit is –log10[(mg/L)/(1000*MW)]
$LC_{50}FM$	3.47	■ 96-hour fathead minnow 50 percent lethal concentration. ■ The unit is –log10[(mg/L)/(1000*MW)]
$LC_{50}DM$	4.303	■ 48-hour daphnia magna 50 percent lethal concentration. ■ The unit is –log10[(mg/L)/(1000*MW)]

9. Tox21 pathway

Property	Value	Decision	Comment
NR-AhR	0.301	🟠	■ Aryl hydrocarbon receptor ■ Category 1: actives ; ■ Category 0: inactives; ■ The output value is the probability of being active.
NR-AR	0.832	🔴	■ Androgen receptor ■ Category 1: actives ; ■ Category 0: inactives; ■ The output value is the probability of being active.
NR-AR-LBD	0.144	🟢	■ Androgen receptor ligand-binding domain ■ Category 1: actives ; ■ Category 0: inactives; ■ The output value is the probability of being active.
NR-Aromatase	0.432	🟠	■ Category 1: actives ; ■ Category 0: inactives; ■ The output value is the probability of being active.
NR-ER	0.991	🔴	■ Estrogen receptor ■ Category 1: actives ; ■ Category 0: inactives; ■ The output value is the probability of being active.
NR-ER-LBD	0.833	🔴	■ Estrogen receptor ligand-binding domain ■ Category 1: actives ; ■ Category 0: inactives; ■ The output value is the probability of being active.
NR-PPAR-gam ma	0.001	🟢	■ Peroxisome proliferator-activated receptor gamma ■ Category 1: actives ; ■ Category 0: inactives; ■ The output value is the probability of being active.
SR-ARE	0.782	🔴	■ Antioxidant response element ■ Category 1: actives ; ■ Category 0: inactives; ■ The output value is the probability of being active.

SR-ATAD5	0.255	●	■ ATPase family AAA domain-containing protein 5<br>■ Category 1: actives ;<br>■ Category 0: inactives;<br>■ The output value is the probability of being active.
SR-HSE	0.022	●	■ Heat shock factor response element<br>■ Category 1: actives ;<br>■ Category 0: inactives;<br>■ The output value is the probability of being active.
SR-MMP	0.812	●	■ Mitochondrial membrane potential<br>■ Category 1: actives ;<br>■ Category 0: inactives;<br>■ The output value is the probability of being active.
SR-p53	0.511	●	■ p53, a tumor suppressor protein<br>■ Category 1: actives ;<br>■ Category 0: inactives;<br>■ The output value is the probability of being active.

10. Toxicophore Rules

Property	Value	Comment
Acute Toxicity Rule	0	■ 20 substructures;<br>■ acute toxicity during oral administration
Genotoxic Carcinogenicity Rule	0	■ 117 substructures;<br>■ carcinogenicity or mutagenicity
NonGenotoxic Carcinogenicity Rule	0	■ 23 substructures;<br>■ carcinogenicity through nongenotoxic mechanisms
Skin Sensitization Rule	2 alerts	■ 155 substructures;<br>■ skin irritation
Aquatic Toxicity Rule	2 alerts	■ 99 substructures;<br>■ toxicity to liquid(water)
NonBiodegradable Rule	1 alerts	■ 19 substructures;<br>■ non-biodegradable
SureChEMBL Rule	0	■ 164 substructures;<br>■ MedChem unfriendly status
FAF-Drugs4 Rule	1 alerts	154 toxic substructures from FAF-Drug4
